# Human Melanoma-Cell Metabolic Profiling: Identification of Novel Biomarkers Indicating Metastasis

**DOI:** 10.3390/ijms21072436

**Published:** 2020-03-31

**Authors:** Mariangela Kosmopoulou, Aikaterini F. Giannopoulou, Aikaterini Iliou, Dimitra Benaki, Aristeidis Panagiotakis, Athanassios D. Velentzas, Eumorphia G. Konstantakou, Issidora S. Papassideri, Emmanuel Mikros, Dimitrios J. Stravopodis, Evagelos Gikas

**Affiliations:** 1 Section of Pharmaceutical Chemistry, Department of Pharmacy, School of Health Sciences, National and Kapodistrian University of Athens (NKUA), 15701 Athens, Greece; mrgkosm@gmail.com (M.K.); katerinail@pharm.uoa.gr (A.I.); dbenaki@pharm.uoa.gr (D.B.); arispan@pharm.uoa.gr (A.P.); 2 Section of Cell Biology and Biophysics, Department of Biology, School of Science, National and Kapodistrian University of Athens (NKUA), 15701 Athens, Greece; aigiann@biol.uoa.gr (A.F.G.); tveletz@biol.uoa.gr (A.D.V.); ipapasid@biol.uoa.gr (I.S.P.); 3 Harvard Medical School, Massachusetts General Hospital Cancer Center (MGHCC), Charlestown, MA 021004, USA; ekonstantakou@mgh.harvard.edu

**Keywords:** biomarker, cancer, melanoma, metabolomics, metastasis, MS, NMR

## Abstract

Melanoma is the most aggressive type of skin cancer, leading to metabolic rewiring and enhancement of metastatic transformation. Efforts to improve its early and accurate diagnosis are largely based on preclinical models and especially cell lines. Hence, we herein present a combinational Nuclear Magnetic Resonance (NMR)- and Ultra High Performance Liquid Chromatography-High-Resolution Tandem Mass Spectrometry (UHPLC-HRMS/MS)-mediated untargeted metabolomic profiling of melanoma cells, to landscape metabolic alterations likely controlling metastasis. The cell lines WM115 and WM2664, which belong to the same patient, were examined, with WM115 being derived from a primary, pre-metastatic, tumor and WM2664 clonally expanded from lymph-node metastases. Metabolite samples were analyzed using NMR and UHPLC-HRMS. Multivariate statistical analysis of high resolution NMR and MS (positive and negative ionization) results was performed by Principal Component Analysis (PCA), Partial Least Squares-Discriminant Analysis (PLS-DA) and Orthogonal Partial Least Squares-Discriminant Analysis (OPLS-DA), while metastasis-related biomarkers were determined on the basis of VIP lists, S-plots and Student’s t-tests. Receiver Operating Characteristic (ROC) curves of NMR and MS data revealed significantly differentiated metabolite profiles for each cell line, with WM115 being mainly characterized by upregulated levels of phosphocholine, choline, guanosine and inosine. Interestingly, WM2664 showed notably increased contents of hypoxanthine, myo-inositol, glutamic acid, organic acids, purines, pyrimidines, AMP, ADP, ATP and UDP(s), thus indicating the critical roles of purine, pyrimidine and amino acid metabolism during human melanoma metastasis.

## 1. Introduction

Melanoma is a cancer that originates from melanocytes, the specialized melanin-producing cells that mainly reside in the skin [1]. Incidence of skin cancer, the 3rd most common human malignancy, is increasing in a progressive rate for western populations, with melanoma being one of its most common forms. While early diagnosis, combined with an appropriate treatment, significantly enhances survival, advanced metastatic melanoma is particularly difficult to treat, showing only 5% long-term survival rate [2]. Hence, further in-depth investigation on melanoma progression and metastasis mechanisms is considered as crucial, in order to accomplish early-stage detection and more efficient treatment.

A malignant hallmark of cancer is cell’s ability to obtain the necessary nutrients from low-content environments, and to productively consume them for maintaining viability and generating new biomass. Tumorigenesis, therefore, depends on rewiring of cellular metabolism, as a direct or indirect consequence of oncogenic mutations disrupting its integrity [3]. Monitoring of metabolic alterations in cancer cells can be implemented through the state-of-the-art metabolomics technology that employs cutting-edge analytical instrumentation, and high resolution techniques, also providing complementary information to genomic and proteomic profiles [4].

Metabolomics has greatly contributed to cancer research, unveiling complex biological mechanisms and deregulated pathways, leading to the discovery of novel biomarkers and personalized treatment schemes [5,6,7]. Specifically, cell metabolomics, despite the demanding sample processing, offer a direct insight in metabolic reprogramming, while it is cost-effective, ethically acceptable and of minimum biological variability [8,9].

Recent metabolomics studies on melanoma have employed either NMR- or MS-based analytical techniques. Melanocytes and various melanoma cell lines of dissimilar metastatic potentials and incomparable mutational signatures were explored, using GC-MS and Direct Infusion MS (DI-MS), confirming glucose and glutamine switched metabolisms, and revealing considerably perturbed alanine and aspartic acid pathways [10,11]. Melanoma cells have also been examined by employing NMR analysis, which underlined the enhanced glutaminolysis and oxidative phosphorylation, as well as the deregulated choline and lipid metabolism [12,13]. WM115 and WM2664 cell metabolisms have been studied using intact cells, or after extraction, showing that glucose accumulation and phospholipid composition differ significantly between the two cell types [14,15].

In the present study, the cell lines WM115 (primary tumor) and WM2664 (lymph-node metastases) that have derived from the same patient, and thus carry comparable and metastasis-related mutational signatures, were thoroughly examined, conducting an untargeted NMR- and UHPLC-HRMS-mediated metabolomics analysis, in order to obtain a better understanding of the underlying biochemistry characterizing these cancer-cell, metastasis-dependent, types. Besides the novel information produced, combination of these two analytical methodologies provides the unambiguous verification of metabolites [7] being essentially engaged in advanced melanoma metastasis. The statistical analysis comparatively employed for each cell-type’s metabolic landscape unearthed significantly differentiated metabolite profiles between pre-metastatic (WM115) and metastatic (WM2664) melanoma cells. WM115 cells were mainly characterized by elevated contents of phosphocholine, choline, guanosine and inosine, whereas WM2664 cells were mapped with notably upregulated levels of hypoxanthine, myo-inositol, glutamic acid and organic acids, as well as AMP, ADP, ATP and UDP(s), strongly suggesting the critical contribution of purine, pyrimidine and amino acid metabolism to human melanoma metastasis.

## 2. Results

### 2.1. NMR

#### 2.1.1. Metabolite Identification

**Figure 1:**
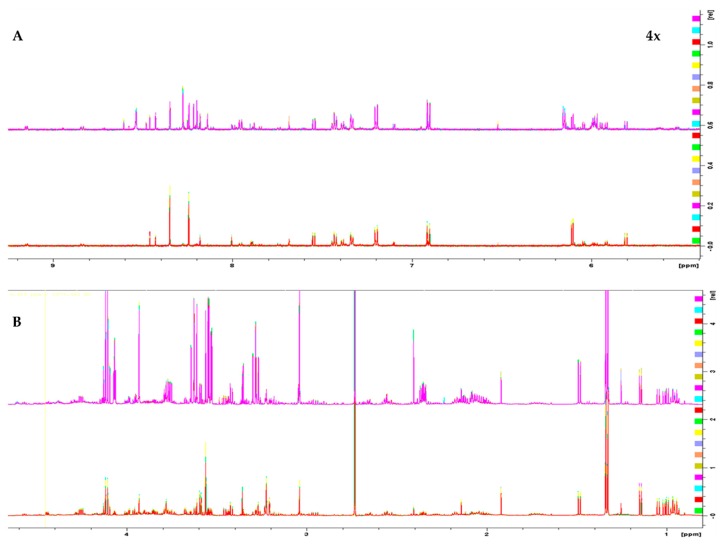
^1^H 1D NMR spectra of: (**A**) the aromatic region and (**B**) the aliphatic region of the WM2664 metastatic (*n* = 10, top) and WM115 pre-metastatic cell group (*n* = 10, bottom). The metastatic group exhibits notable upregulation of most metabolites. Aromatic region was magnified (4×) compared to the aliphatic one, in order to aid visual inspection.

^1^H 1D NMR spectra of the two melanoma cell lines, WM115 and WM2664, are shown in Figure 1. The Chenomx NMR Suite v. 8.4 and the HMDB online library were used for identification of the extracted metabolites via NMR spectroscopy [16]. The human melanoma-derived metabolites are described in Table S1, while a representative WM2664 annotated spectrum is given in Figure S2. A total of 50 metabolites were identified across all samples, with 39 of them being recognized to significantly differ between the two melanoma cell lines in the statistical tests.

#### 2.1.2. Chemometric Analysis of NMR Data

**Figure 2:**
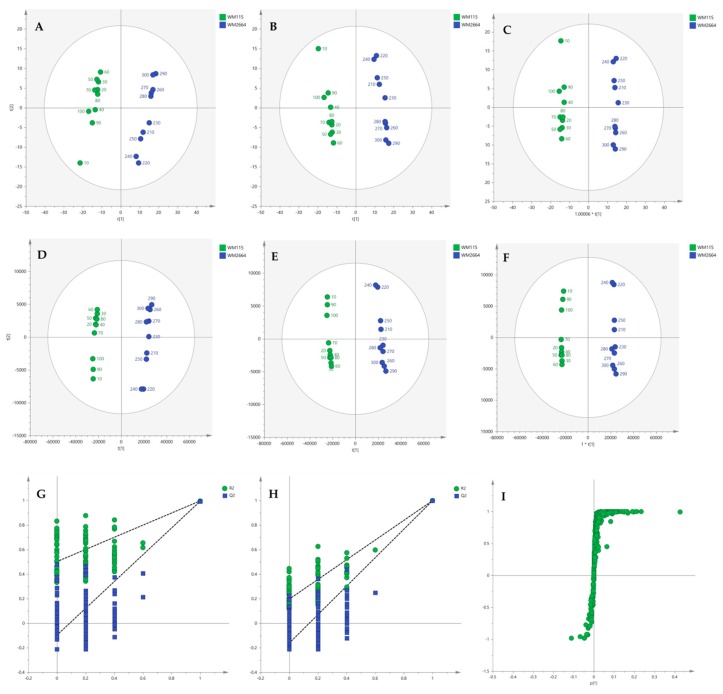
(**A**) PCA, (**B**) PLS-DA and (**C**) OPLS-DA score plots in UV scaling, and (**D**–**F**) the corresponding scores plots in Pareto scaling of NMR data. Permutation tests of PLS-DA models (**G**) in UV scaling και (**H**) in Pareto scaling. (**I**) S-plot from OPLS-DA model.

Multivariate statistical analysis was performed, by employing SIMCA P 14.1. In the first step, Principal Components Analysis (PCA) was applied, in order to visualize the obtained data, with the aid of recognizing possible trends and locating possible outliers. Both Unit Variance (UV) and Pareto scaling exhibited clear separation between the two cell lines, WM115 and WM2664, without the presence of outlying values (UV scaling: R^2^X(cum) = 0.712 and Q^2^(cum) = 0.637; Pareto scaling: R^2^X(cum) = 0.953 and Q^2^(cum) = 0.936) (Figure 2A,D). Next, Partial Least Squares-Discriminant Analysis (PLS-DA) was engaged, using also the UV and Pareto scaling (Figure 2B,E). Analysis resulted to clear separation of the two cell groups, with generated models showing very good predictive ability (UV scaling: R^2^X(cum) = 0.711, Q^2^(cum) = 0.994, 2 components; Pareto scaling: R^2^X(cum) = 0.953, Q^2^(cum) = 0.998, 2 components). Validation of PLS-DA models was performed, allowing of 100 permutations, in order to assess the prediction power of the classification (Figure 2G,H). Results proved that obtained generated models were not produced randomly and, therefore, they were considered as valid. Finally, Orthogonal Partial Least Squares-Discriminant Analysis (OPLS-DA) led to an equally good separation of the two melanoma cell lines, in both scaling methods (UV scaling: R^2^X(cum) = 0.711, Q^2^(cum) = 0.994; Pareto scaling: R^2^X(cum) = 0.953, Q^2^(cum) = 0.998) (Figure 2C,F).

**Figure 3:**
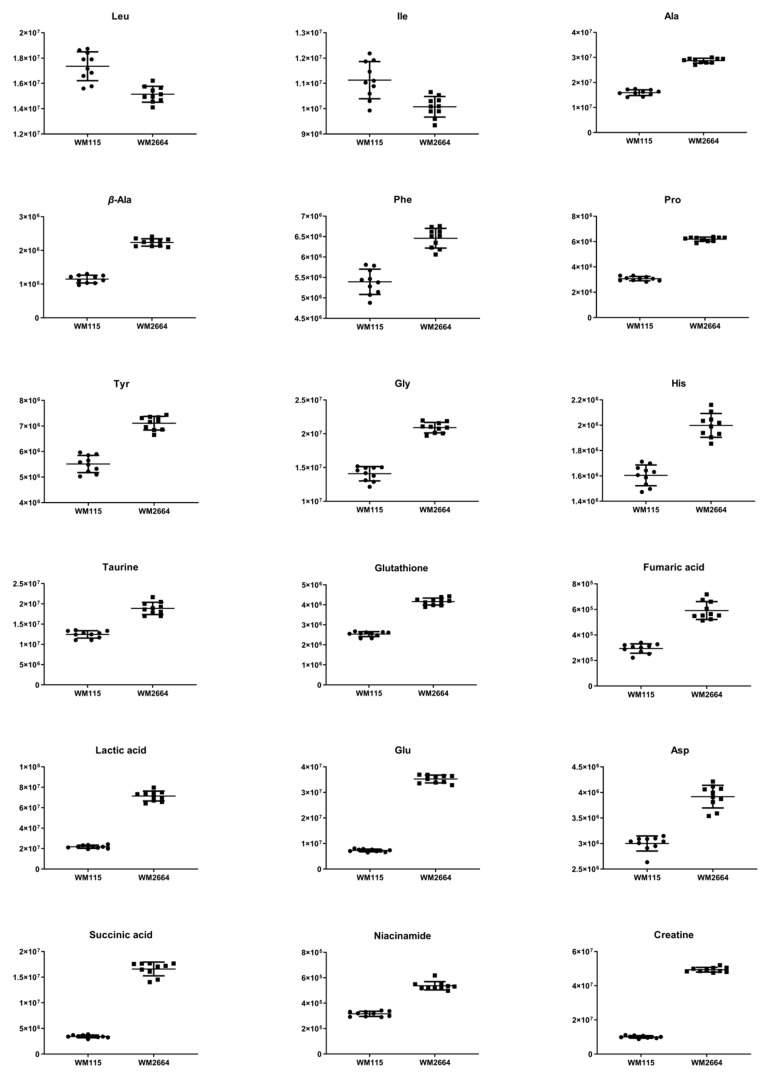

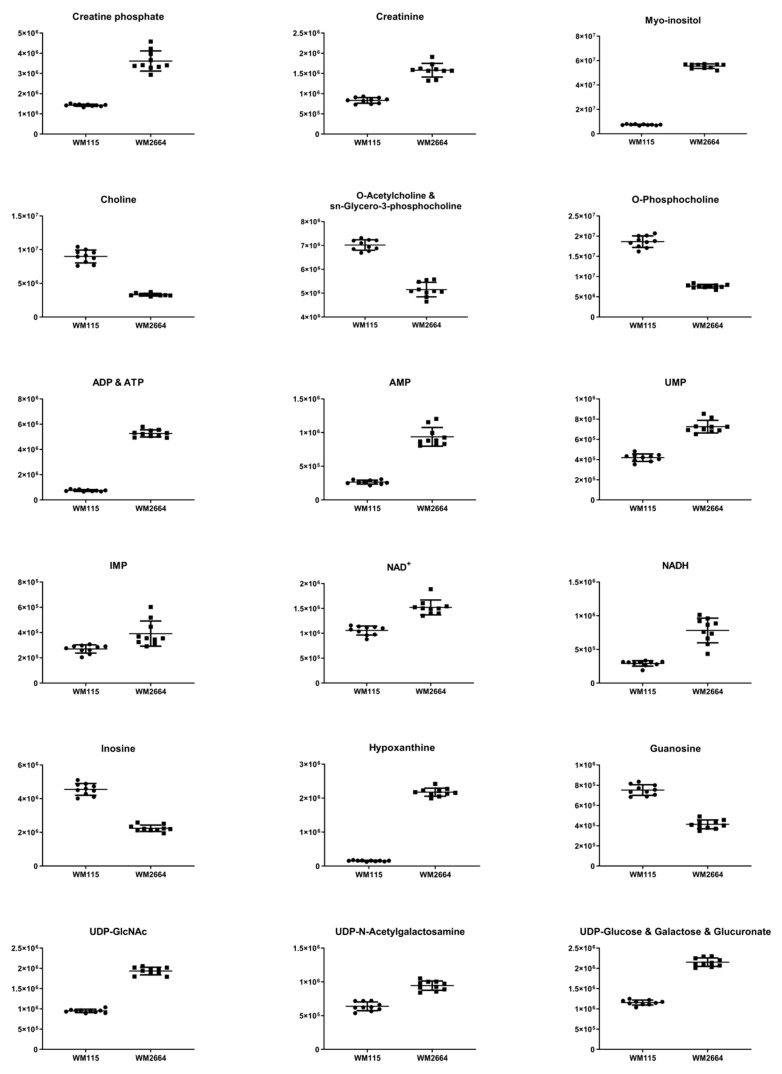
Boxplots for melanoma-originated metabolites (AUC ≥ 0.9) that have been herein identified in NMR data.

Distant features towards the extremes were selected from the S-plot (OPLS-DA model with Pareto scaling) (Figure 2I), while those exhibiting Variable Importance in Projection (VIP) > 1 were considered as responsible for the separation between the two cell groups (Table 1). The identified features, together with their corresponding statistical-test information and boxplots, are shown in Table 2 and Figure 3, respectively, with most of them presenting statistically significant differences between the two melanoma cell groups, WM115 and WM2664, herein examined.

**Figure 4:**
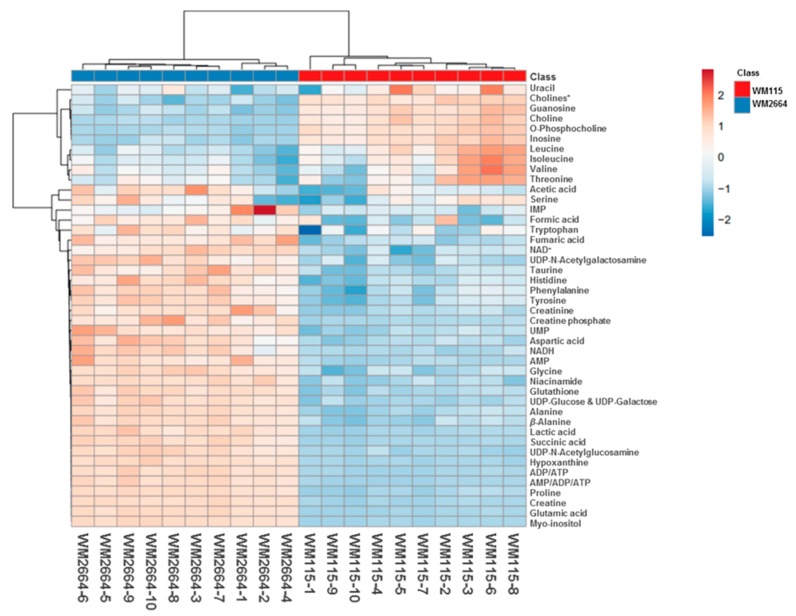
Presentation of NMR-identified, melanoma-derived, metabolites, in a heatmap form. * O-Acetylcholine and sn-Glycero-3-phosphocholine.

Rapid and reliable identification of human pathology-discrimination metabolites is an issue of major importance in metabolomics, and, thus, heat-maps have the advantage of a direct and systemic overview of the data-matrix analyzed. Hence, NMR data heat-map illustrates an excellent sample clustering and indicates a collection of novel mechanistic biomarkers for human pre-metastatic and metastatic melanoma (Figure 4), with significant value in disease prognosis, diagnosis and therapy in the clinic.

**Figure 5:**
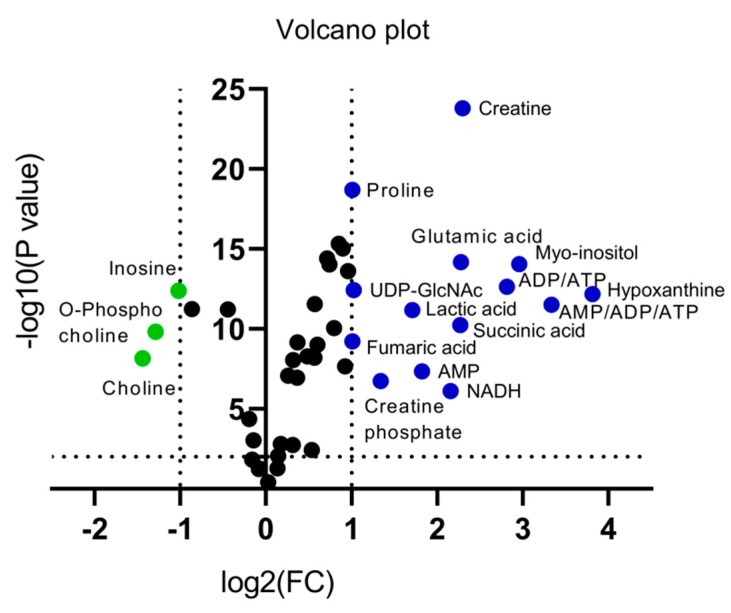
Volcano plot of human melanoma metabolites. Fold Change (FC) < 0.5, or Fold Change (FC) > 2 and *p* < 10^−5^ are observed for metabolites indicated in color.

An alternative visualization of the statistical results of “omics” data is the Volcano plot, which allows the identification of metabolites that exhibit the strongest combination of Fold Change (FC) (x) and statistical significance (*p* value). The Volcano diagram is two-dimensional, with the *x*-axis representing the variation ratio of each metabolite (log2FC) and the *y*-axis the *p* value obtained from the corresponding statistical test t (-log10P) [17,18] (Table 2). Metabolites with the most significant contribution to the separation of the two melanoma cell lines, WM115 and WM2664, are highlighted in color, with those in the left segment being elevated in WM115 (green) and those being identified in the right segment presenting an increase in WM2664 (blue) (Figure 5).

**Figure 6:**
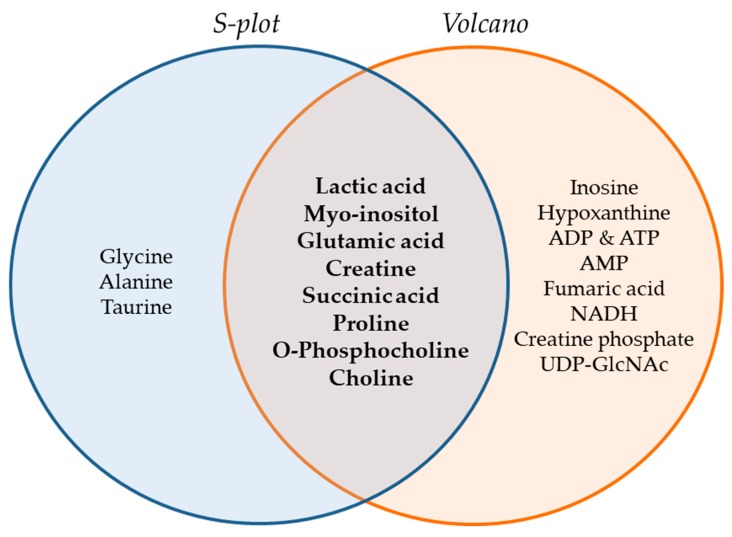
Venn diagram of melanoma metabolites that are related to metastasis. Important metabolites are highlighted (bold fonts) at the interphase of S-plot and Volcano plot.

The Venn diagram of Figure 6 summarizes the metabolites that were found to be important for separation of the two cell lines, WM115 and WM2664, by multivariate (S-plot, VIPs) and univariate statistical analysis (Volcano plot). Metabolites that emerged from both statistical approaches are presented at the intersection of both sets, and, thus, may serve as mechanistic biomarkers, with strong prognostic, diagnostic and therapeutic potential, for human advanced melanoma undergoing metastasis.

### 2.2. UHPLC-HRMS/MS

**Figure 7:**
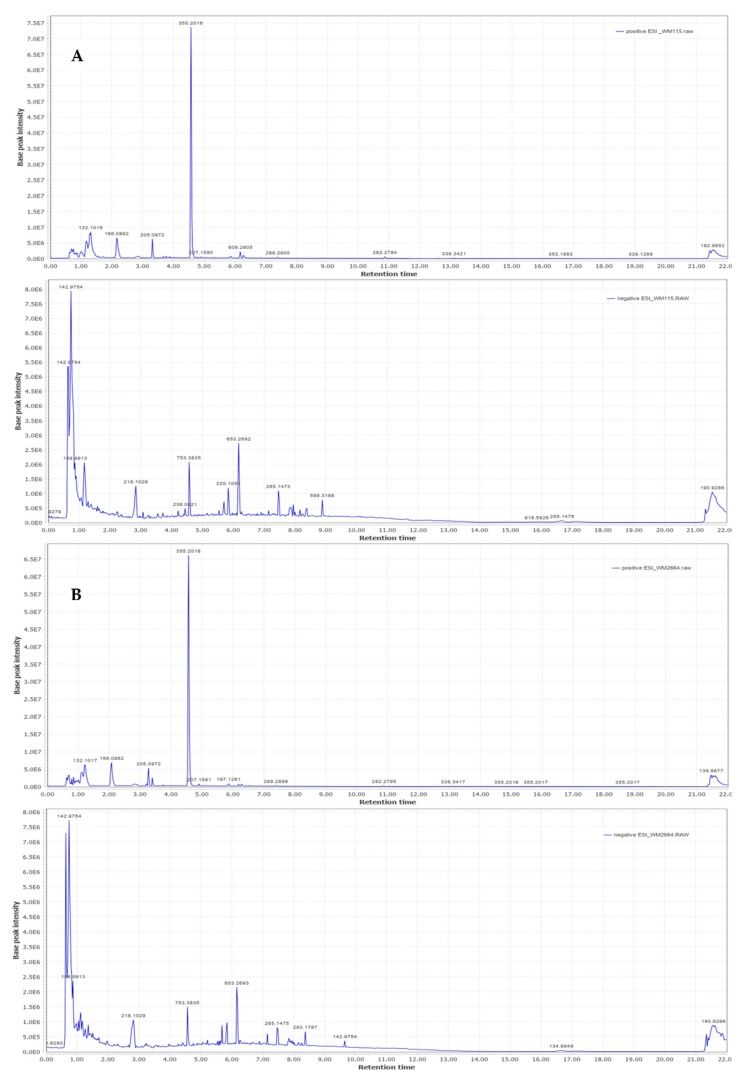
Typical base peak intensity chromatograms of: (**A**) WM115 and (**B**) WM2664 melanoma cells, for positive (upper panels) and negative (bottom panels) ion mode.

Representative base peak intensity chromatograms of WM2664 cells, in the positive and negative ion mode, are shown in Figure 7.

#### 2.2.1. MS Spectra Processing

The results obtained as a CSV file, after pre-processing of the mass spectra with MZmine 2.31, were evaluated based on the presence of internal standards in all samples and the repeatability of their integration regions, in order to select the appropriate algorithm parameters. For positive Electrospray Ionization (ESI), precursor ions of all three internal standards were detected, whereas in negative ionization the precursor ions for the two internal standards and the Cl adduct for 2-aminophenol were detected. Subsequently, the data were submitted into SIMCA 14.1 and PCA analysis was performed, for all the 20 distinct samples (10 per cell line) and the 9 replicates of Quality Control (QC) sample (Figure S1). Scores plot exhibits that repetitions of the QC sample do not follow clustering, therefore suggesting systematic error due to instrumental drift, a serious complication that is often encountered in mass spectrometry. Thereby, the QC-robust spline batch correction (QC-RSC) method of normalization, as implemented within the online platform MetaX, was suitably applied, in order to reduce systematic error between measurements, so that only biological differences between examined samples were highlighted [19,20]. Selection of the normalization algorithm is a critical parameter in data analysis. To evaluate results for normalization algorithms and find the most appropriate one, they were, next, analyzed by PCA, using SIMCA 14.1. The more closely arranged the QC samples are shown, the more accurate the normalization method is considered. The Cross-contribution Compensating Multiple standard Normalization (CCMN) algorithm presented the best results in clustering of control samples and was, therefore, chosen as the most suitable normalization method. Figure S1A,B describes the diagram of PCA in UV scaling, for negative ionization, before and after normalization.

#### 2.2.2. Chemometric Analysis of MS Data

**Figure 8:**
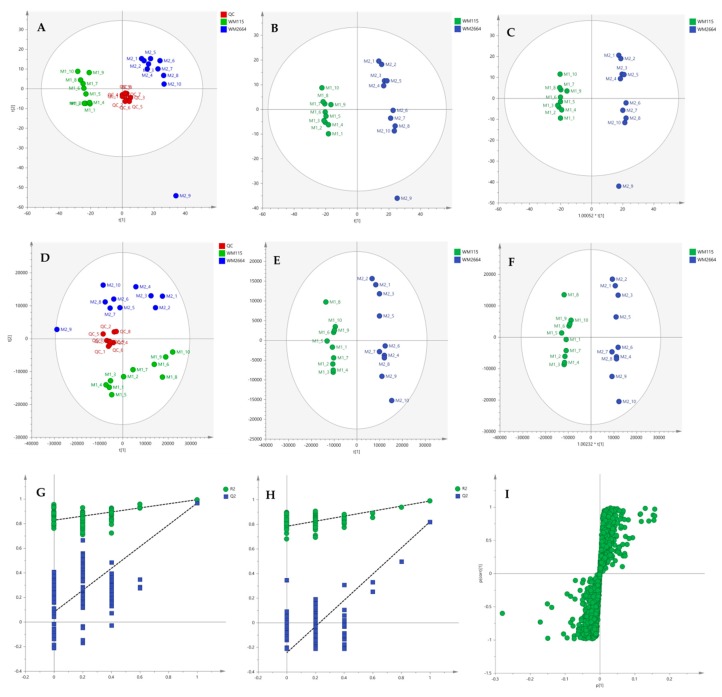
(**A**) PCA, (**B**) PLS-DA and (**C**) OPLS-DA scores plots in UV scaling, and (**D**–**F**) the corresponding scores plots in Pareto scaling of Mass Spectrometry (MS) data, in negative ion mode. Permutation tests of PLS-DA models (**G**) in UV και (**H**) in Pareto scaling. (**I**) S-plot from OPLS-DA model.

**Figure 9:**
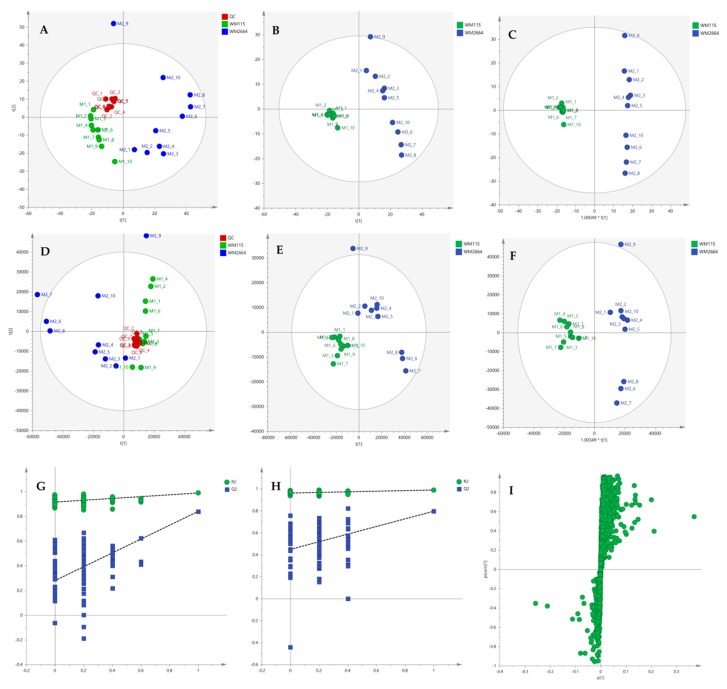
(**A**) PCA, (**B**) PLS-DA and (**C**) OPLS-DA scores plots in UV scaling, and (**D**–**F**) the corresponding scores plots in Pareto scaling of MS data, in positive ion mode. Permutation tests of PLS-DA models (**G**) in UV και (**H**) in Pareto scaling. (**I**) S-plot from OPLS-DA model.

After the initial data pre-processing, multivariate analysis (PCA, PLS-DA and OPLS-DA) was applied, in order to explore the underlying metabolic pathways that are mechanistically related to melanoma metastasis. The PCA generated models are presented as scores plot in Figure 8A,D, for the negative (UV and Pareto scaling, respectively) and in Figure 9A,D, for the positive ionization mode (UV and Pareto scaling, respectively). For PCA analysis, R^2^ and Q^2^ were 0.502 and 0.207, for UV, and 0.447 and −0.009, for Pareto scaling, respectively, in the negative ion mode, whereas in the positive ionization, R^2^X(cum) and Q^2^(cum) were 0.433 and 0.13, for UV, and 0.585 and 0.016, for Pareto scaling, respectively. The 9th sample of WM2664 cell group appears as an outlier (negative and positive ionization), as it can be observed in PCA diagrams, but it is not rejected, as being within the acceptable 5% of the total samples, at the 95% confidence level. Based on the PCA approach, the overall UHPLC-HRMS analysis, after data normalization, is considered reliable for statistical evaluation, since the control samples follow a close clustering pattern.

By applying the supervised PLS-DA methodology, a clear separation between the WM115 and WM2664 cell groups was observed, as it can be nicely described in the corresponding diagrams for negative and positive ion modes, in Figure 8 and Figure 9. The Q^2^(cum) value was 0.966 for the negative ionization, while, for the positive ionization, was 0.865, choosing the 2 main components in UV scaling. The OPLS-DA supervised model used, for both negative (R^2^X(cum) and Q^2^(cum) were 0.427 and 0.958, respectively) and positive ionization (0.369 and 0.856, respective values), showed excellent fitting and predictive capacity.

Furthermore, to confirm the validity of the obtained model, permutation testing was conducted. Not any random model was produced, exhibiting higher R^2^ and Q^2^ values than the unpermutated, respective, ones (Figure 8G,H and Figure 9G,H), thus proving the validity of the obtained PLS-DA model. Next, in order to identify those features that contribute the most to the separation, the S-plot was employed, based on the OPLS-DA model, for both ionization modes, engaging SIMCA 14.1. S-plot features that lie remotely from the main-data cluster were selected, as they are considered to contribute the most, with high reliability, to the group separation (Figure 8I and Figure 9I). Features in the lower-left quadrant correspond to the WM115 cell group, while features in the upper-right quadrant belong to the WM2664 cell group. To verify, the statistically obtained from S-plot, results, t-test and False Discovery Rate (FDR) values [21,22] were calculated, using Graphpad Prism 7. A Q value of 5% was used, for both positive and negative ionization modes. As potential biomarkers were accepted the ones whose S-plot features proved to significantly differ between cell groups, according to t-test and FDR calculations. Due to the large number of features that met the aforementioned criteria (86 for negative and 17 for positive ionization), a smaller range of features was selected for identification (12 for negative and 15 for positive ionization) (Table S2).

#### 2.2.3. Identification of Selected MS Features

The R statistical package xMSannotator [23,24], as well as the METLIN [25] and HMDB [16] databases, were suitably employed to identify the selected features. Presence of adducts and MS/MS fragment ions, consistent with METLIN and HMDB data for each particular metabolite, were examined, and the isotope ratio was also calculated, for every identified feature. The following scoring scale was used to evaluate the reliability of each assignment: two points for each parent ion or adduct or pseudo MS/MS fragment, 1.5 points for each MS/MS fragment (as it has been acquired in low-resolution mode, using parallel scans to Linear Ion Trap) and 0.5 points for isotopic ratio showing Relative Standard Deviation < 30% (RSD < 30%) when compared to the theoretically computed one by the Thermo Xcalibur Qual Software Browser [26]. Assignments with a sum of less than or equal to four points were considered to be sufficiently reliable. Table 3 presents the melanoma-derived metabolites identified in negative and positive ionization modes.

#### 2.2.4. Targeted Metabolite Screening Based on Biochemical Pathways

Identification data, derived from both NMR Spectroscopy and Mass Spectrometry, revealed metabolites critically implicated in major (canonical), or alternative (non-canonical), biochemical pathways. Therefore, targeted screening for the remaining metabolites of these pathways, as well as for other selected metabolites that may essentially contribute to human carcinogenesis, could provide important additional information regarding the human skin oncometabolome. This approach primarily focused on “rare” amino acids, urea cycle, arginine metabolism and cycle of Lynen (ketone bodies). Presence of parent ions, and their respective Na, K and NH_4_ adducts, together with corresponding dimers, for all the selected metabolites, were carefully considered in both ionization modes.

Targeted screening was performed with the MZmine 2.31 software and the parameters used are listed in Table S3. Metabolites identified in the negative (Table 4), or positive (Table 5) ionization mode were processed with the same manner as the one chosen for those of untargeted screening.

**Figure 10:**
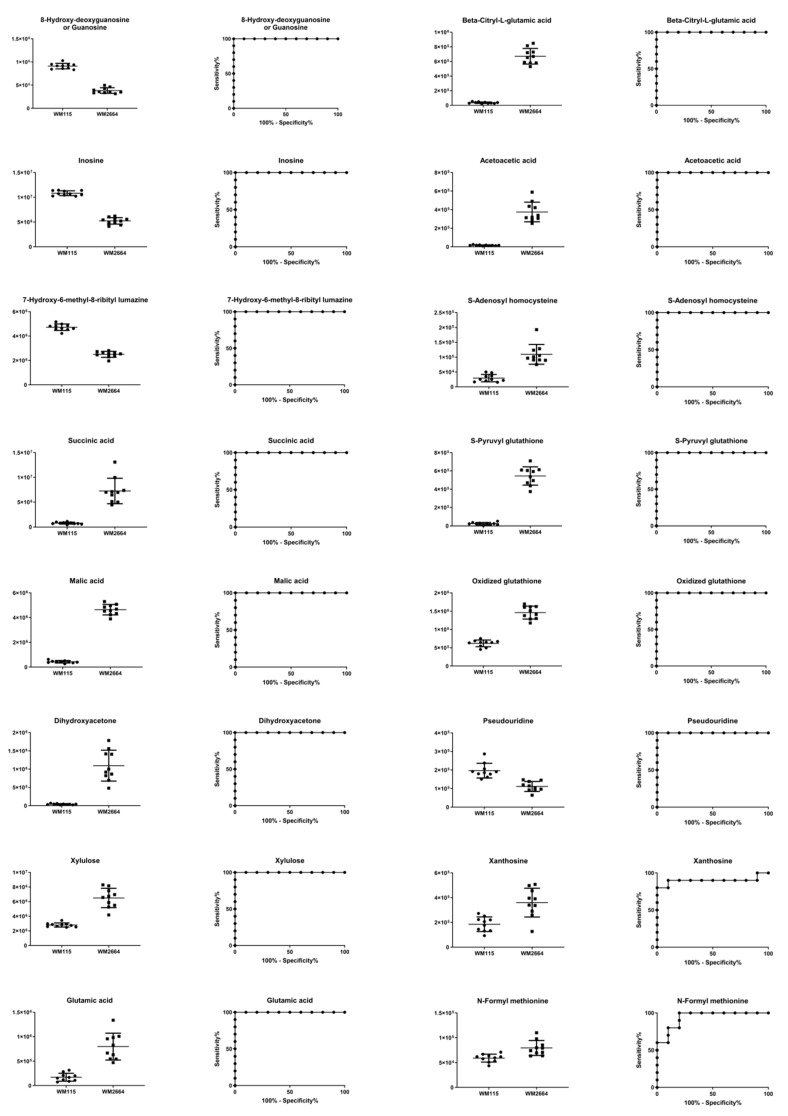
Boxplots and ROC curves, for identified MS features, in negative ion mode, with AUC > 0.8.

**Figure 11:**
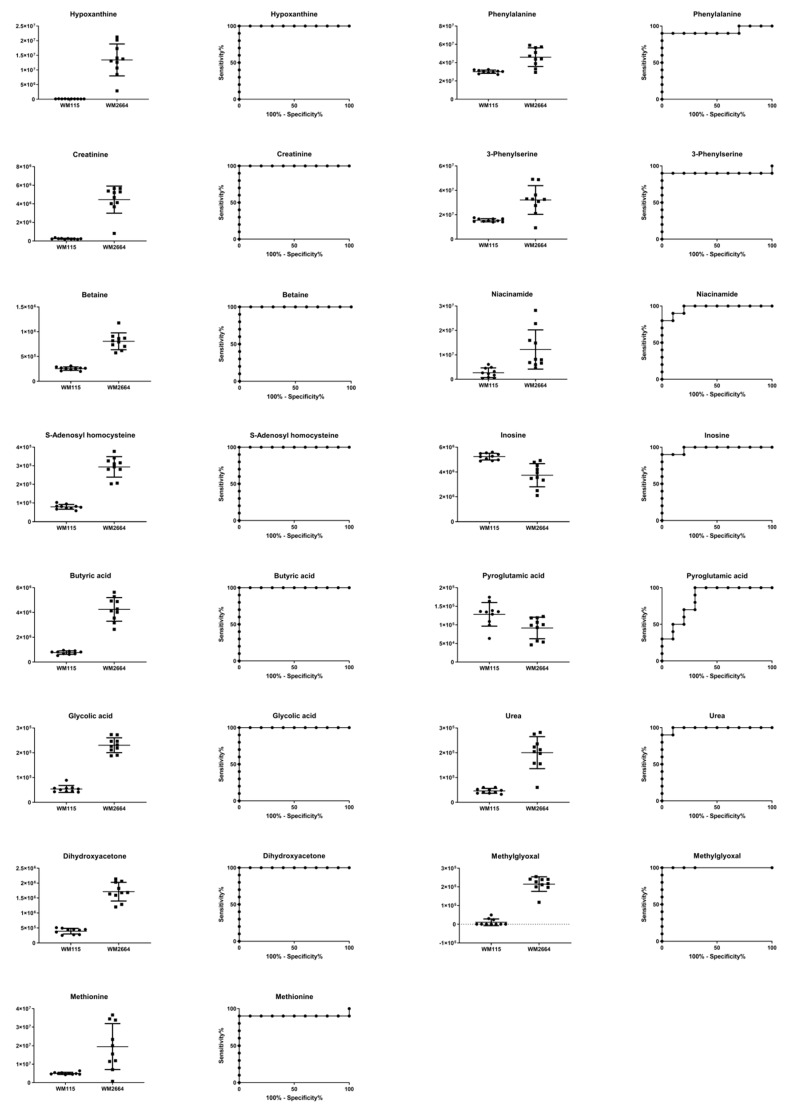
Boxplots and ROC curves, for identified MS features, in positive ion mode, with AUC > 0.8.

Boxplots and ROC curves, for the most significant metabolites recognized from MS data, are shown in Figure 10 (negative ionization) and Figure 11 (positive ionization).

## 3. Discussion

Metabolic rewiring is a hallmark of malignant transformation in several types of cancer [3,27,28]. Tumor microenvironment, which is often not sufficiently vascularized, compels tumor cells to oxygen lack and nutrient deprivation, orchestrating metabolic changes that promote survival and growth in stressful conditions. Rapid proliferation increases the demand for energy supply that is required for macromolecular biosynthesis, as well as synthesis of amino acids, nucleotides and lipids.

One of the major alterations detected in the present study is the upregulated glycolysis and the increased production of lactic acid, likely derived from pyruvic acid to prevent its oxidation in mitochondria even in the presence of oxygen. This phenomenon was, first, described in 1920 by Otto Warburg and is called the “Warburg effect” [29,30]. Alternatively, enhanced glycolysis may likely result in accumulation of the methylglyoxal (MG) cytotoxic metabolite that is spontaneously formed by the degradation of two glycolytic intermediates [31]. Hence, cancer cells, in order to avoid MG-induced self-destruction, and as a survival strategy, upregulate the glyoxalase-dependent enzymatic machinery to remove MG and convert it into D-lactic acid, thus causing its cellular accumulation [31]. Interestingly, overexpression of glyoxalases has been observed in human melanoma [32]. Beyond this metabolic reprogramming, and since glucose is converted almost quantitatively to lactic acid, the TCA (tricarboxylic acid) cycle functions, regardless of oxygen presence, in the reverse direction, using glutamine as an alternative carbon source [10]. Elevated glycolysis and glutaminolysis could explain the absence of both glucose and glutamine metabolites from the two cell lines, WM115 and WM2664, but most importantly the significant increase in lactic acid (FC_NMR_ = 3.27) and glutamic acid (FC_NMR_ = 4.84; FC_MS_ = 5.81), as well as in the TCA cycle intermediates fumaric acid (FC_NMR_ = 2.01) and succinic acid (FC_NMR_ = 4.82; FC_MS_ = 4.04) [11], in the metastatic melanoma cell line WM2664. A rise in the (metabolite-linker) alanine content [10] from implementation of these two processes is expected, since alanine aminotransferase catalyzes the transfer of an amino group from glutamic to pyruvic acid, with alanine and oxoglutaric acid being the reaction products. Glutamine-derived oxoglutaric acid supplies the TCA cycle with additional carbon, while the excess nitrogen is secreted in the form of alanine. In addition to the approximately 2-fold (x) increase of the detected alanine (FC_NMR_ = 1.80) in the metastatic cells, a similar elevation was also observed for proline (FC_NMR_ = 2.01), whose enhanced generation is a characteristic metabolic change in melanoma [33,34], with rather glutamine than arginine serving as a key metabolic precursor. Synthesis of arginine in melanoma is often suppressed due to lack of argininosuccinate synthetase (ASS) [35] (an enzyme involved in the urea cycle), which could justify the absence of arginine from both cell lines.

The glycolysis flux may also be diverted from the intermediate 3-phosphoglyceric acid to serine synthesis, a process that seems upregulated in melanoma [36]. A significant percentage of serine content (FC_NMR_ = 1.02) produced is converted to glycine (FC_NMR_ = 1.49), the uptake, synthesis and consumption of which have been previously associated with tumorigenesis [37]. Creatine is formed from glycine by two consecutive reactions and, in turn, can be phosphorylated into phosphocreatine, ultimately generating adenosine triphosphate (ATP) [38]. The elevated concentration of creatine (FC_NMR_ = 4.91) and phosphocreatine (FC_NMR_ = 2.53) in WM2664 cells mainly reflects their essential roles in energy storage required under the increased metabolic rates of tumor cells. In addition to synthesis of ATP, these two metabolites are also involved in its trafficking from the internal mitochondrial membrane to the cytoplasm, thus acting as energy distribution regulators [38,39]. However, ATP, the most important molecule in the cell-energy network and homeostasis, is predominantly formed during the glycolysis process, as well as by oxidative phosphorylation in mitochondria. The energy requirements of cancer cells are clearly increased and they rise proportionally to the rate of tumor growth, hence justifying the significantly elevated ADP/ATP contents (FC_NMR_ = 7.04) in the WM2664 metastatic melanoma cells (as compared to the WM115 cell line of reference).

Enhanced glycolysis in melanoma can result in increased glucose flow to the hexosamine biosynthetic pathway (HBP), with UDP-N-acetylglucosamine (UDP-GlcNAc) being the final product, a high-energy donor of the GlcNAc group of protein substrates, which was herein proved as an elevated critical metabolite in the WM2664 cell line (FC_NMR_ = 2.04). It has been previously reported that the levels of β-1,6-branched N-glycans are often increased in malignant tumors and this rise is closely related to tumor progression [40,41]. Interestingly, incubation of B16 melanoma cells with GlcNAc upregulates β-1,6-branched oligosaccharide levels in proportion to the elevation of intracellular UDP-GlcNAc [42].

Another glycolysis product herein shown to be notably elevated in WM2664 cells is myo-inositol (FC_NMR_ = 7.76). This metabolite is intrinsically synthesized from glucose in three steps: (a) glucose is phosphorylated by hexokinase, (b) produced glucose 6-phosphate is, next, converted to myo-inositol 1-D-phosphate (by the MIPS synthase) and (c) myo-inositol 1-D-phosphate is finally dephosphorylated towards free myo-inositol [43]. Myo-inositol plays an essential role in regulating cell volume and osmotic pressure, while in vitro and in vivo NMR studies unveil correlation with tumor-cell density (its concentration is often increased) [9]. Myo-inositol addition to B16 melanoma cells, having been pre-treated with LiCl, partially inhibited the anti-proliferative and morphological effect of LiCl on these cells [44].

Taurine, a metabolite that is biosynthesized via the cysteinesulfinic acid pathway [45], exhibits metabolic activity and role similar to myo-inositol ones in cancer, while it seems increased in WM2664 metastatic melanoma cells (FC_NMR_ = 1.52). Although it is thought to possess anti-oxidant properties, its elevated contents may highlight the importance of oxidative stress in metastatic melanoma and in disrupted balance of metabolic pathways [46].

The metabolite that demonstrated the most striking difference between the two cell lines, WM115 and WM2664, was hypoxanthine, whose concentration was measured at least 14-fold (x) higher in WM2664 (FC_NMR_ = 14.05; FC_MS_ = 94.26), as compared to WM115 (reference) cells. Metabolic studies for gastric cancer and melanoma have described higher plasma and tissue levels, due to higher tumor-cell proliferation rates, conditions under which hypoxanthine acts as a substrate and source of nitrogen [11,47]. Hypoxanthine is formed via the Purine Biosynthesis Pathway (PBP) [48] that is characterized by the conversion of ribose 5-phosphate to inosine monophosphate (FC_NMR_ = 1.45), which is, then, dephosphorylated to inosine, the major hypoxanthine precursor molecule. Thereby, the remarkably increased levels of hypoxanthine are tightly linked to the significantly decreased contents of inosine in the WM2664 metastatic melanoma cells (FC_NMR_ = 0.49; FC_MS_ = 0.48). It seems that primary melanoma favors the metabolic route of inosine monophosphate to guanosine (FC_MS_ = 0.42), a molecule that is notably elevated in the WM115 cells and has been suggested to serve as biomarker [14].

Metabolites that are also presented with a statistically significant increase in the primary melanoma WM115 cell group were the choline (FC_MS_ = 0.37), phosphocholine (FC_MS_ = 0.41) and glycerophosphocholine (FC_MS_ = 0.73), which are key components of cell membranes [9]. Abnormal choline metabolism has proved as a critical metabolic feature being tightly associated with aberrant membrane synthesis and tumorigenesis. In the majority of malignant cases, choline is extensively metabolized to phosphocholine [49]. The observed reduction of phosphocholine levels in WM2664 cells likely derives from augmentation of lipid metabolism in metastatic melanoma [50] and/or from acidic environment(s) of the tumor limiting its formation [51].

In contrast to the rest of the herein identified amino acids, leucine (FC_NMR_ = 0.87), isoleucine (FC_NMR_ = 0.91) and, to a lower extent, valine (FC_NMR_ = 0.95) were presented with decreased contents in the WM2664 cell line. Their degradation leads to production of acetyl-coenzyme A, which is, then, oxidized via the TCA cycle. Branched-chain amino acids (BCAAs) are essential components of proteins, mainly due to their hydrophobicity, and, therefore, in oncogenic conditions of increased protein synthesis and energy requirement, their consumption and oxidation are often intensified [52]. Although results from other metabolic cancer studies converge to the experimental reduction of BCAAs [11,49], differentiation of their levels has not proved consistent [50].

Interestingly, a metabolite that also exhibited a decrease in the WM2664 metastatic melanoma cells, and was identified by non-targeted MS analysis, was the 7-hydroxy-6-methyl-8-ribityl lumazine [53] (FC_MS_ = 0.53), which seems to be implicated in the action of melanosomal tyrosinase [54]. Melanosome constitutes an organelle for melanin synthesis, storage and transfer, but, despite the number of reports, its mechanistic direct correlation with melanoma and metastasis requires further investigation. Since 7-hydroxy-6-methyl-8-ribityl lumazine has been previously proposed as biomarker for prostate cancer [55], it could similarly serve as melanoma biomarker, with strong prognostic, diagnostic and therapeutic value.

**Figure 12:**
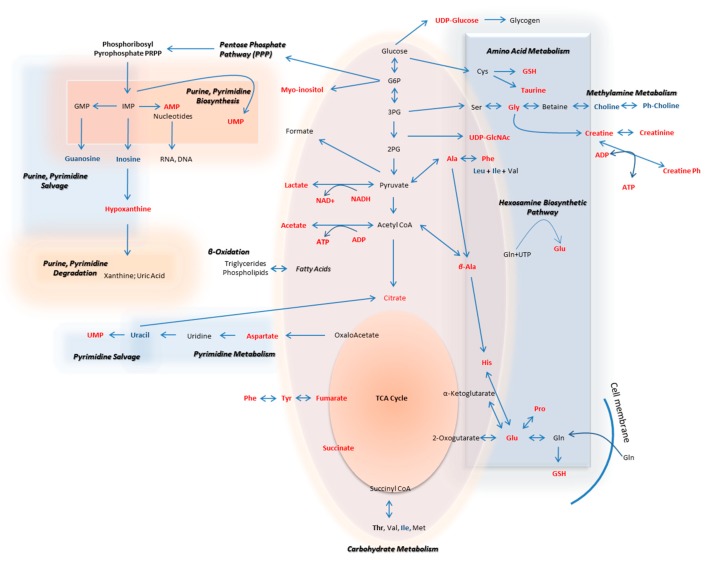
Flow-diagram showing pathways and cognate metabolites critically involved in the discrimination of primary and metastatic human melanoma cells. Blue: reduced levels (Fold Change < 0.50); Red: increased levels (Fold Change > 2). The metastatic cell group is presented with elevated contents of hypoxanthine, myo-inositol, AXP (X: D, or T), UDPs, amino acids and organic acids, and decreased levels of guanosine, inosine and cholines, thus indicating the perturbed purine, pyrimidine and amino acid metabolism during metastasis in melanoma.

The metabolic pathways that are herein unearthed to most likely control metastasis in human melanoma are illustrated in Figure 12 (metabolites with statistically significant increase in the WM2664 metastatic melanoma cells are shown in red, while the ones significantly reduced in blue).

## 4. Materials and Methods

### 4.1. Chemicals and Reagents

DMEM culture medium was purchased from Thermo Fischer Scientific Inc.-Life Technologies-Gibco (Waltham, MA, USA). Phosphate-Buffered Saline (PBS), and other cell-culture media and reagents were provided from Merck Millipore-Biochrom AG (Merck KgAa, Darmstadt, Germany). Deuterium oxide (D_2_O) was purchased from Deutero GmbH (Kastellaun, Germany). Trimethylsilyl propionic (TSP) (used as internal standard in NMR analysis), methanol, acetonitrile, and chloroform were purchased from Sigma-Aldrich-Merck (Darmstadt, Germany). Formic acid was obtained from PanReac Applichem (Darmstadt, Germany). Chemicals were of analytical grade and dissolved in ultrapure water (ddH_2_O). For the MS analysis, reserpine, yohimbine and 2-aminophenol were used as internal standards (Sigma-Aldrich, Steinheim, Germany). All chemicals were of LC-MS (Liquid Chromatography-Mass Spectrometry) grade.

### 4.2. Cell Lines and Culture Conditions

The human melanoma cell lines WM115 (primary tumor) and WM2664 (lymph-node metastases), which derived from the same (female; 58 years old) patient, were purchased from ECACC/Sigma-Aldrich (Munich, Germany), and they were cultured in 1× DMEM medium, being supplemented with fetal bovine serum, L-glutamine, sodium pyruvic, sodium bicarbonate, non-essential amino acids, penicillin and streptomycin, at 37 °C, 5% CO_2_, and > 95% humidity.

### 4.3. Cell Collection and Storage

For each cell line, 10^7^ melanoma cells, grown in high-density (high-confluence) cultures, produced samples of the required high quantity and quality for metabolomics studies. Culture dishes were placed on ice, medium was aspirated and cells were washed twice with ice-cold 1× PBS to remove residual medium traces. Ice-cold 1× PBS, up to the minimum necessary volume, to cover the culture plate, was added, for immediate quenching of cell metabolism. Specifically, cells were harvested through a mild scrapping process and the suspensions (1× PBS and (adherent, and suspended) cells) were centrifuged at 550× *g* for 5 min at 4 °C. Supernatants were aspirated and cell pellets were stored at −80 °C, for further metabolomics processing. Ten samples from each cell line, WM115 and WM2664, were simultaneously analyzed.

### 4.4. Extraction Protocol

Metabolites were extracted according to Wu’s stepwise protocol [56]. Briefly, 200 μL of ice-cold methanol and 200 μL of chloroform were added to the frozen, metabolically quenched, cells, and the suspension was sonicated for 5 min on ice. Next, 180 μL of ddH_2_O was added and vortexing for 1 min followed. Processed samples remained on ice for 15 min, centrifuged at 16,000 rpm for 20 min at 4 °C and the upper aqueous phase together with the lower organic phase were collected. The whole procedure was repeated once more (except from the sonication step) and the two phases, after being suitably collected, were added to the corresponding ones derived from the first extraction step.

### 4.5. Sample Preparation

Polar extracts were dried in a centrifuge evaporator, and reconstituted in phosphate buffer pH = 7.4 (containing TSP as internal standard and NaN_3_ as preservative) and deuterated water (700 μL, 10:90 (*v*/*v*), Buffer:D_2_O), for the preparation of NMR samples. 100 μL of each NMR sample were dried under vacuum and washed twice with water:acetonitrile, 95:5 (*v*/*v*), in order to exchange the deuterated protons for MS analysis. Dried samples were reconstituted in 100 μL of water:acetonitrile, 95:5 (*v*/*v*), containing 1% of internal standards. A pooled sample was prepared by mixing aliquots (16 μL) of each individual sample and was used as Quality Control (QC) sample, for mass analysis performance control.

### 4.6. NMR Analysis

All NMR experiments were performed using the NMR Bruker AVANCE III 600 MHz Spectrometer at 300 K, equipped with a PABBI z-gradient probe. After the optimization of acquisition conditions, a fully automated procedure was accomplished through the ICONNMR program suite (TOPSPIN 2.1 version, Bremen, Germany), controlling a B-ACS 60 system (Bruker BioSpin GmbH, Bremen, Germany). ^1^H 1D NMR spectra were acquired with 256 scans of 64 k data points, over a spectral width of 12,335.5 Hz. The acquisition time was 2.65 s, the relaxation delay was 9.0 s and the mixing time was 0.01 s. Solvent suppression of residual water was achieved using the standard NOESY (Nuclear Overhauser Effect Spectroscopy) pre-saturation pulse sequence (noesygppr1D, Bruker library). 2-D J-resolved spectra were acquired with 4 scans of 8 k data points over a spectral width of 10,000 Hz, resulting in an acquisition time of 0.4 s; the relaxation delay was 1 s. For a selected sample, WM2664 cell line, homonuclear (Total Correlation Spectroscopy, TOCSY) and heteronuclear (Heteronuclear Single Quantum Coherence Distortionless Enhancement by Polarization Transfer, HSQC-DEPT 135) 2D spectra were recorded to aid the unambiguous assignment of metabolites. The TOCSY experiment was performed with 128 scans while 2 k data points were collected over a spectral width of 9615.4 Hz. The acquisition time was 0.1 s and the relaxation delay was 1.5 s. HSQC-DEPT 135 experiment was performed for a spectral width of 9615.4 Hz for proton frequencies and 27,164 Hz for ^13^C; 320 scans were collected.

### 4.7. MS Analysis

The UHPLC-ESI-LTQ ORBITRAP Discovery System (Thermo Fisher Scientific, Rockford, IL, USA) was used for data acquisition, and the Xcalibur 2.1 software was employed for the subsequent visualization, and the initial MS analysis. For chromatographic separation of the metabolome, a C_18_ Hypersil Gold column (100 × 2.1 mm, 1.7 μm) (Thermo Fisher Scientific, Rockford, IL, USA) and a pre-column Waters VanGuard (5 × 2.1 mm, 1.7 μm) were used, with the mobile phase being consisted of solvent A: 0.1% formic acid in ddH_2_O (*v*/*v*) and solvent B: acetonitrile. Gradient elusion was performed as follows: 0–16 min, 98%A:2%B; 16–22 min, 2%A:98%B; 22 min, 100% B for positive and 0–17 min, 98%A:2%Β; 17–22 min, 2%A:98%Β; 22–24 min, 98%A:2%B for negative ion mode. Column temperature was kept at 40 °C throughout all experiments, while auto-sampler temperature was set at 8 °C. A QC sample was being injected every 5 consecutive samples, under the same experimental conditions. The injection volume was 5 μL, and the mass-scan range was set at 100–1000 m/z in the positive and 115–1000 m/z in the negative ion mode. The conditions for HRMS in each ionization mode were set as shown in Table S4A. Analysis was performed using the Fourier Transform Mass Spectrometry mode of the LTQ Orbitrap (FTMS), in the full-scan ion mode, applying a resolution of 30,000, while acquisition of mass spectra was performed in every case using the centroid mode. Data-dependent acquisition (DDA) capability has been also engaged, allowing for MS/MS fragmentation of the 3 most intense ions.

### 4.8. Data Pre-Processing

#### 4.8.1. NMR

Pre-processing of the NMR spectra (data reduction and bucketing) was conducted using the AMIX Ver. 3.9.12 suite (Analysis of MIXtures, Bruker BioSpin, GmbH). ^1^H NMR spectra from 9.40 to 0.60 ppm were segmented into 0.02 and 0.001 ppm bucket widths. Regions, including residual water signal (δ 5.50–4.631 ppm) and contaminations observed in blank samples (δ 1.260–1.241 ppm and δ 2.250–2.221 ppm), were excluded from the analysis [57,58,59]. When normalization to total intensity is performed, alterations of major metabolites may cause biased results for low concentration metabolites [60]. Hence, in order to avoid biased results, and since there was no need to correct for biological or analytical variability, we decided not to perform any normalization. NMR bins, prior to multivariate analysis, were scaled to Unit Variance (UV) and Pareto and the results were compared.

#### 4.8.2. MS

Mass Spectrometry (MS)-data pre-processing [19] was performed by employing MZmine 2.31 [61], Microsoft Excel 2010 and the MetaX [62] online platform. Initially, raw data were introduced into MZmine 2.31, via which noise was filtered, while peaks were detected, using the ADAP Chromatogram Builder algorithm [63], and extracted-ion chromatograms (EICs) were obtained for each sample tested. Next, peak de-convolution was carried out with the Wavelets ADAP algorithm, a continuous wavelet transform (CWT)-based peak-detection algorithm, which uses shape information based on calculated coefficients and, consequently, reduces false positives [64]. Subsequently, isotopic distribution search was performed, with the “isotopic-peak grouper” and chromatographic peaks being aligned via employment of the Join Aligner algorithm, according to their elution time. Finally, after filling in the gap entries, the CSV file was exported. The final Table was edited in Microsoft Excel 2010, using the ”concatenate“, ”round“ and ”transpose“ commands. Data were normalized based on QC samples, prior to statistical analysis, with the QC-RSC algorithm of the MetaX platform (Table S4B).

### 4.9. Statistical Analysis

Principal Component Analysis (PCA), Partial Least Square-Discriminant Analysis (PLS-DA) and Orthogonal Partial Least Square-Discriminant Analysis (OPLS-DA) were herein engaged, as implementation via the SIMCA P 14.1 software (Umetrics, Umea, Sweden). Chemometric models were validated, using permutation tests with 100 random permutations. In order to pinpoint the most influential variables for group separation, selection of those with high-magnitude and -reliability of the S-plot derived from the OPLS-DA approach and the corresponding Variable Importance in Projection (VIP) values were taken into account, following a combinational fashion. The GraphPad Prism 7 software was used for the calculation of AUROC (Area Under the Receiver Operating Characteristic) curves and following a series of univariate statistical tests for each identified metabolite ((a) normality test (D’ Agostino and Pearson), (b) outlier identification test (Grubbs) and (c) heteroscedasticity test (F-test)) were conducted prior to Student’s t-test, or Welch test. Multiple hypothesis testing for false positive results, were minimized with Bonferroni-Dunn and, the less strict, False Discovery Rate (FDR) corrections. Fold Change (x) (FC = (mean_WM2664_)/(mean_WM115_)) and z-score ((mean_WM2664_ − mean_WM115_)/SD_WM115_) were also calculated, using Microsoft Excel 10.

## 5. Conclusions

Melanoma accounts for more than 60% of all deaths associated with skin cancer, thus necessitating its early and accurate detection to improve treatment outcomes. Given the pivotal role of metabolic reprogramming in cancer initiation and progression, identification of specific metabolic patterns that can differentiate pre-metastatic from metastatic tumor cells may serve as a valuable biochemical tool that carries strong prognostic, diagnostic and therapeutic potential.

Using Nuclear Magnetic Resonance (NMR) Spectroscopy and High-Resolution Mass Spectrometry (HRMS) Coupled with Ultra High Performance Liquid Chromatography (UHPLC-HRMS), as part of a non-targeted metabolic approach based on cell activity, metabolomes of the human melanoma cell lines WM115 (primary) and WM2664 (metastatic), both derived from the same patient, were herein profiled. Next to pre-processing of the original data, a number of critical metabolites were identified, via suitable and successful engagement of the ChenomX NMR software, and the HMDB and METLIN libraries for MS and MS/MS spectra, while univariate and multivariate statistical analyses were performed using the SIMCA 14.1 and GraphPad software Prism 7 platforms.

A collection of 50 metabolites were detected through the NMR analysis, 40 of which presented a statistically significant difference between the pre-metastatic WM115 and metastatic WM2664 cells of human melanoma. Moreover, 30 out of 36 metabolites proved to differ significantly between the two respective metastatic states (Table 3, Table 4 and Table 5) via the UHPLC-HRMS analysis (10 common metabolites with NMR). Differentially synthesized (or consumed/degraded), secreted and/or sequestered metabolites are components of a diverse set of fundamental metabolic pathways, such as the glucose catabolism, glutamine catabolism and choline metabolism, which seem to critically contribute to the survival and growth of advanced melanoma cells. Interestingly, hypoxanthine, ATP/ADP/AMP, myo-inositol, creatine, glutamic acid, succinic acid, NADH, lactic acid, creatine phosphate, UDP-N-acetylglucosamine, proline and fumaric acid were found significantly elevated in WM2664, whereas choline, o-phosphocholine, inosine, guanosine and leucine were presented with notably reduced contents in the same cell line, and, therefore, they could all be considered as powerful tumor biomarkers, carrying strong prognostic, diagnostic and therapeutic values for the metastatic form(s) of human advanced melanoma(s). Mapping of novel metabolic landscapes in melanoma cells that grow in vitro and bear distinct metastatic activities is expected: (a) to illuminate our knowledge and understanding regarding the Molecular Biology of advanced melanoma, and (b) to open new therapeutic windows for the metastatic disease form(s) in the clinic.

## Figures and Tables

**Table 1:** Melanoma metabolites selected from S-plot (OPLS-DA model in Pareto scaling), as the most influential in group separation, and their corresponding VIP values.

Metabolites Elevated (↑) in WM115			Metabolites Elevated (↑) in WM2664		
Name	S-plot (δ)	VIP	Name	S-plot (δ)	VIP
o-Phosphocholine	3.23	2.18	Lactic acid	1.33	8.36
Choline	3.21	1.34	Myo-inositol	3.55	4.60
Inosine	8.35	0.91	Myo-inositol	4.07	4.15
Inosine	6.11	0.87	Myo-inositol	3.63	4.12
Leucine	0.97	0.83	Lactic acid	4.11	3.83
Isoleucine	1.27	0.70	Myo-inositol	3.53	3.76
			Myo-inositol	3.29	3.29
			Myo-inositol	3.61	3.23
			Glutamic acid	2.35	3.17
			Creatine	3.93	3.16
			Creatine	3.03	2.92
			Creatine	3.05	2.92
			Myo-inositol	3.65	2.66
			Glutamic acid	3.77	2.40
			Myo-inositol	3.27	2.35
			Succinic acid	2.41	2.21
			Glutamic acid	2.37	2.13
			Glutamic acid	2.07	2.12
			Lactic acid	4.13	2.07
			Proline	3.35	2.06
			Glutamic acid	3.79	1.90
			Glutamic acid	2.13	1.86
			Glutamic acid	2.09	1.79
			Glutamic acid	3.75	1.77
			Glutamic acid	2.05	1.77
			Lactic acid	4.09	1.75
			Glutamic acid	2.15	1.67
			Glycine	3.57	1.66
			Glutamic acid	2.33	1.65
			Alanine	1.49	1.64
			Taurine and Proline ^1^	3.43	1.62
			Myo-inositol	3.31	1.61
			Glutamic acid	2.11	1.51

^1^: At δ 3.43 there is contribution from both Taurine and Proline resonances.

**Table 2:** Statistical tests for melanoma-derived metabolites that were being identified in NMR data.

Metabolite (*bold: AUC ≥ 0.9 and adjusted p < 0.05*)	Outliers (*Grubbs*)	Normality test (*D’ Agostino and Pearson*)	F-Test Significantly Different Variance (*p < 0.05*)	Welch’s t-Test (*p Value*)	t-Test (*p Value*)	Bonferroni–Dunn (*Adjusted p Value*)	z-Score	Fold Change (*x*)	AUC	Tendency
**Alanine**	0	√	no		4.77E−16	2.16E−14	11.09	1.80	1	↑WM2664
***β*-Alanine**	0	√	no		2.38E−14	1.08E−12	9.52	1.95	1	↑WM2664
**Aspartic acid**	0	√	yes	1.12E−07		5.00E−06	11.50	1.29	1	↑WM2664
**Glutamic acid**	0	√	yes	6.68E−15		3.02E−13	52.93	4.84	1	↑WM2664
**Glutathione**	0	√	no		3.98E−15	1.80E−13	13.12	1.64	1	↑WM2664
**Glycine**	0	√	no		2.74E−12	1.21E−10	6.43	1.49	1	↑WM2664
**Histidine**	0	√	no		9.08E−09	4.09E−07	4.81	1.25	1	↑WM2664
**Isoleucine**	0	√	no		0.000911	0.0409	−1.43	0.91	0.9	↑WM115
**Leucine**	0	√	no		4.22E−05	0.00189	−1.94	0.87	0.96	↑WM115
**Phenylalanine**	0	√	no		8.58E−08	4.00E−06	3.45	1.20	1	↑WM2664
**Proline**	0	√	no		1.96E−19	9.00E−18	18.52	2.01	1	↑WM2664
Serine	0	√	no		0.394	1	0.39	1.02	0.6	
**Taurine**	0	√	no		9.89E−10	4.45E−08	7.08	1.52	1	↑WM2664
Threonine	0	√	no		0.0585	1	−0.73	0.94	0.73	
Tryptophan	0	√	no		0.00159	0.0720	1.34	1.13	0.88	
**Tyrosine**	0	√	no		6.85E−10	3.11E−08	4.74	1.29	1	↑WM2664
Valine	0	√	no		0.0554	1	−0.74	0.95	0.74	↑WM115
**Creatine**	0	√	no		1.59E−24	7.20E−23	54.01	4.91	1	↑WM2664
**Creatine phosphate**	0	√	yes	1.8E−07		8.00E−06	41.57	2.53	1	↑WM2664
**Creatinine**	0	√	yes	2.19E−08		9.90E−07	10.70	1.90	1	↑WM2664
Acetic acid	0	√	no		0.00870	0.392	1.47	1.10	0.83	
Formic acid	0	√	yes	0.00180		0.0810	1.37	1.24	0.85	
**Fumaric acid**	0	√	no		6.03E−10	2.70E−08	8.10	2.01	1	↑WM2664
**Lactic acid**	0	√	yes	6.79E−12		3.06E−10	32.51	3.27	1	↑WM2664
**Succinic acid**	0	√	yes	5.63E−11		2.52E−09	49.00	4.82	1	↑WM2664
**Choline**	0	√	yes	7.02E−09		3.15E−07	−5.93	0.37	1	↑WM115
**sn-Glycero-3-phosphocholine & *O*-Acetylcholine**	0	√	no		6.01E−12	2.70E−10	−8.45	0.73		↑WM115
***O*-Phosphocholine**	0	√	yes	1.52E−10		8.55E−17	−7.68	0.41	1	↑WM115
**AMP**	0	√	yes	4.52E−08		2.00E−06	23.14	3.54	1	↑WM2664
**ADP & ATP**	0	√	yes	2.36E−13		1.08E−11	64.99	7.04	1	↑WM2664
**Guanosine**	0	√	no		5.86E−12	2.66E−10	−6.45	0.55	1	↑WM115
**Hypoxanthine**	0	√	yes	6.81E−13		3.06E−11	131.73	14.05	1	↑WM2664
**Inosine**	0	√	no		4.06E−13	1.85E−11	−6.63	0.49	1	↑WM115
IMP	0	√	yes	0.00381		0.171	3.74	1.45	0.97	↑WM2664
**NAD^+^**	0	√	no	5.17E−09		2.34E−07	4.63	1.40	1	↑WM2664
**NADH**	0	√	yes	7.67E−07		0.000035	21.87	4.46	1	↑WM2664
Uracil	0	√	no		0.0149	0.671	−0.97	0.89	0.82	↑WM115
**UDP-*N*-Acetylglucosamine**	0	√	yes	3.66E−13		1.66E−11	23.04	2.04	1	↑WM2664
**UDP-Galactose & Glucose & Glucuronic acid**	0	√	no		9.18E−16	4.14E−14	16.21	1.86	1	↑WM2664
**UDP-*N*-Acetylgalactosamine**	0	√	no		6.31E−09	2.84E−07	4.73	1.48	1	↑WM2664
**UMP**	0	√	no		8.74E−11	3.91E−09	8.33	1.73	1	↑WM2664
**Myo-inositol**	0	√	yes	8.72E−15		3.91E−13	101.96	7.76	1	↑WM2664
**Niacinamide**	0	√	no		9.13E−15	4.10E−13	10.58	1.67	1	↑WM2664

**Table 3:** Selected ^1^ melanoma metabolites, together with their corresponding detected parent ions, adducts and MS/MS fragments, theoretical and experimental m/z, ppm error, retention time (t_R_), isotopic ratio Relative Standard Deviation (RSD) (%), *p* value, Fold Change (FC) and reliability score.

Name	Adducts	Theoretical *m*/*z*	Experimental *m*/*z*	ppm Error	t_R_ (min)	Adducts	MS/MS	Isotopic Ratio RSD (%)	*p* Value	FC	Trust Level	ESI
8-Hydroxy-deoxyguanosine or Guanosine	M-H	282.084	282.0839	−1.86	1.14	M+FA-H	0	<30%	1.51E−13	0.42	4.5	(-)
Malic acid	M-H	133.014	133.0146	3.05	0.74	-	114.91	<30%	3.33E−11	10.74	4	(-)
7-Hydroxy-6- methyl-8-ribityl lumazine	M+K-2H	365.051	365.0495	−2.66	1.17	-	267.06	<30%	1.81E−13	0.53	4	(-)
beta-Citryl-l- glutamic acid	M-H	320.062	320.0621	−0.54	0.85	-	0	<30%	1.29E−08	18.93	2.5	(-)
*S*-pyruvyl glutathione	M+FA-H	422.088	422.0868	−1.66	0.99	-	0	<30%	3.92E−08	22.79	2.5	(-)
Oxidized glutathione	M-H	611.145	611.1432	−2.40	0.99	-	0	<30%	3.43E−09	2.36	2.5	(-)
3-Phenylserine	M + H	182.0812	182.0812	0.44	0.94	-	136, 165, 147	<30%	0.00153	2.07	7	(+)
Methionine	M+H	150.0582	150.0582	−0.99	0.86	-	133, 104	<30%	0.00495	3.89	5.5	(+)
Phenylalanine	M+H	166.0862	166.0862	−0.13	2.05	-	120, 149	<30%	0.000736	1.53	5.5	(+)
Niacinamide	M+H	123.055	123.0550	−2.02	1.05	-	80	<30%	0.00448	4.56	4	(+)

^1^: AUC = 1, for metabolites identified in negative ion mode and AUC ≥ 0.9, for metabolites identified in positive ion mode.

**Table 4:** Melanoma-derived metabolites ^1^ identified via targeted screening in negative ionization, and their corresponding detected parent ions, adducts and MS/MS fragments, theoretical and experimental m/z, ppm error, retention time (t_R_), isotopic ratio RSD (%), *p* value, Fold Change (FC) and reliability score.

Name	Adducts	Theoretical *m*/*z*	Experimental *m*/*z*	ppm Error	t_R_ (min)	Adducts	MS/MS	Isotopic Ratio RSD (%)	*p* Value	FC	Trust Level
**Inosine**	M-H	267.0730	267.0731	0.21	1.16	M+Cl, M+FA-H, 2M+FA-H, 2M-H	134.93, 134.98, 135.04	<30%	7.95E−15	0.46	13.5
**Succinic acid**	M-H	117.0187	117.0196	7.69	1.11		99, 73	<30%	0.000302	4.04	5.5
Allantoic acid	2M-H	351.1010	351.1008	−0.51	4.54		307.11 (pseudo)	<30%	0.000339	0.67	4.5
**Xanthosine**	M-H	283.0678	283.0678	0.00	1.50	M+FA-H		<30%	8.77E−06	2.02	4.5
**Dihydroxyacetone**	2M-H	179.0550	179.0561	6.14	0.82	M+Cl		ND^2^	0.000297	32.96	4
**S-Adenosyl-** **homocysteine**	M-H	383.1138	383.1139	0.26	0.92			<30%	1.13E−08	36.60	2.5
**Xylulose**	M+K-2H	187.0003	187.0012	5.05	0.82			<30%	1.31E−05	2.39	2.5
N2-Methyl guanosine	M+K-2H	334.0548	334.0553	1.64	3.29			<30%	0.000287	434068.2	2.5
**Xanthurenic acid**	M+Na-2H	226.0116	226.0122	2.65	0.84			ND	0.000459	0.83	2
**Glutamic acid**	M-H	146.0453	146.0460	4.79	0.76			ND	9.3E−07	5.81	2
**Acetoacetic acid**	M+FA-H	147.0300	147.0299	−0.80	0.80			ND	2.67E−07	188.88	2
***N*-Formyl methionine**	M-H	176.0381	176.0385	2.14	3.14			ND	0.00212	1.21	2
**Pseudouridine**	M+FA-H	289.0671	289.0674	1.04	0.95			ND	1.71E−05	0.62	2

^1^: AUC > 0.8, for metabolites in bold, ^2^: not detected.

**Table 5:** Melanoma-originated metabolites ^1^ identified via targeted screening in positive ionization, and their corresponding detected parent ions, adducts and MS/MS fragments, theoretical and experimental m/z, ppm error, retention time (t_R_), isotopic ratio RSD (%), *p* value, Fold Change (FC) and reliability score.

Name	Adducts	Theoretical *m*/*z*	Experimental *m*/*z*	ppm Error	t_R_ (min)	Adducts	MS/MS	Isotopic Ratio RSD (%)	*p* Value	FC	Trust Level
**Inosine**	M+H	269.0880	269.0884	1.31	1.20	M+K, M+Na, 2M+H	136	< 30%	8.77E−14	0.50	10
**Hypoxanthine**	M+H	137.0458	137.0456	−1.53	0.86	M+2K-H, M+K	119, 109	< 30%	2.51E−08	94.26	9.5
**Creatinine**	M+H	114.0662	114.0659	−2.03	0.78	M+2K-H, M+K	86	< 30%	0.000462	6.89	8
**Betaine**	M+K	156.0421	156.0420	−0.41	0.69	M+2K-H		ND ^2^	1.72E−12	3.50	4
Isovaleric acid	2M+ACN+H	246.1700	246.1701	0.41	3.75		85	< 30%	0.000229	0.74	4
**Propionic acid**	2M+ACN+H	190.1074	190.1074	0	3.68	M+ACN+H		ND	2.07E−05	0.46	4
***S*-Adenosyl-homocysteine**	M+H	385.1289	385.1293	0.98	1.02			< 30%	1.21E−09	3.38	2.5
Allantoic acid	2M+H	353.1169	353.1166	−0.68	4.41			< 30%	0.01502	0.83	2.5
**Methylglyoxal**	2M+K	183.0054	183.0054	0	0.76			< 30%	2.12E−09	20.70	2.5
**Butyric acid**	2M+ACN+H	218.1392	218.1388	−1.83	1.48			< 30%	1.22E−12	3.75	2.5
Formic acid	M+2K+H	122.9250	122.9242	−6.22	0.69			< 30%	1.06E−05	1.60	2.5
**Dihydroxy acetone**	M+2K+H	166.9512	166.9507	−3.17	0.71			ND	1.56E−07	2.90	2
Dimethyl arginine	M+H	203.1508	203.1503	−2.59	0.75			ND	2.23E−06	0.17	2
**Pyroglutamic acid**	M+2K+H	205.9616	205.9617	0.44	0.72			ND	0.002998	0.60	2
**Glycolic acid**	2M+NH_4_	170.0659	170.0655	−1.96	1.02			ND	2.99E−12	5.37	2
Hippuric acid	M+ACN+H	221.0921	221.0922	0.42	1.42			ND	1.47E−08	0.05	2
**Urea**	M+2Na-H	105.0035	105.0032	−2.31	0.62			ND	0.000128	2.63	2

^1^ AUC > 0.8, for metabolites in bold, ^2^: not detected.

## Supplementary Materials

Supplementary Materials can be found at https://www.mdpi.com/1422-0067/21/7/2436/s1.

## References

[1] Ali Z., Yousaf N., Larkin J. (2013). Melanoma epidemiology, biology and prognosis. EJC Suppl..

[2] Cummins D.L., Cummins J.M., Pantle H., Silverman M.A., Leonard A.L., Chanmugam A. (2006). Cutaneous Malignant Melanoma. Mayo Clin. Proc..

[3] Ratnikov B.I., Scott D.A., Osterman A.L., Smith J.W., Ronai Z.A. (2017). Metabolic rewiring in melanoma. Oncogene.

[4] Beger R.D. (2013). A Review of Applications of Metabolomics in Cancer. Metabolites.

[5] Nicholson J.K. (2006). Global systems biology, personalized medicine and molecular epidemiology. Mol. Syst. Biol..

[6] Nicholson J.K., Lindon J.C., Holmes E. (1999). “Metabonomics”: Understanding the metabolic responses of living systems to pathophysiological stimuli via multivariate statistical analysis of biological NMR spectroscopic data. Xenobiotica.

[7] Nicholson J.K., Lindon J.C. (2008). Systems biology: Metabonomics. Nature.

[8] Zhang A., Sun H., Xu H., Qiu S., Wang X. (2013). Cell Metabolomics. OMICS.

[9] Griffin J.L., Shockcor J.P. (2004). Metabolic profiles of cancer cells. Nat. Rev. Cancer.

[10] Scott D.A., Richardson A.D., Filipp F.V., Knutzen C.A., Chiang G.G., Ronai Z.A., Osterman A.L., Smith J.W. (2011). Comparative Metabolic Flux Profiling of Melanoma Cell Lines. J. Biol. Chem..

[11] Kim H.-Y., Lee H., Kim S.-H., Jin H., Bae J., Choi H.-K. (2017). Discovery of potential biomarkers in human melanoma cells with different metastatic potential by metabolic and lipidomic profiling. Sci. Rep..

[12] Figueiredo Rodrigues M., Obre E., Melo F., Santos G., Galina A., Jasiulionis M., Rossignol R., Rumjanek F., Amoêdo N. (2015). Enhanced OXPHOS, glutaminolysis and beta-oxidation constitute the metastatic phenotype of melanoma cells. Biochem. J..

[13] Fedele T.A., Galdos-Riveros A.C., Jose de Farias e Melo H., Magalhães A., Maria D.A. (2013). Prognostic relationship of metabolic profile obtained of melanoma B16F10. Biomed. Pharmacother..

[14] Ramachandran G.K., Yeow C.H. (2017). Proton NMR characterization of intact primary and metastatic melanoma cells in 2D & 3D cultures. Biol. Res..

[15] Yu Z., Huang M., Clowers B.H. (2018). Comparative metabolite profiling of a metastatic and primary melanoma cell line using untargeted metabolomics: A case study. Clin. Mass Spectrom..

[16] Wishart D.S., Feunang Y.D., Marcu A., Guo A.C., Liang K., Vázquez-Fresno R., Sajed T., Johnson D., Li C., Karu N. (2018). HMDB 4.0: The human metabolome database for 2018. Nucleic Acids Res..

[17] Cui X., Churchill G.A. (2003). Statistical tests for differential expression in cDNA microarray experiments. Genome Biol..

[18] De Hertogh B., De Meulder B., Berger F., Pierre M., Bareke E., Gaigneaux A., Depiereux E. (2010). A benchmark for statistical microarray data analysis that preserves actual biological and technical variance. BMC Bioinform..

[19] Katajamaa M., Orešič M. (2007). Data processing for mass spectrometry-based metabolomics. J. Chromatogr. A.

[20] Alonso A., Marsal S., Julià A. (2015). Analytical Methods in Untargeted Metabolomics: State of the Art in 2015. Front. Bioeng. Biotechnol..

[21] Benjamini Y., Hochberg Y. (1995). Controlling The False Discovery Rate—A Practical And Powerful Approach To Multiple Testing. J. Royal Statist. Soc. Ser. B.

[22] Benjamini Y., Krieger A.M., Yekutieli D. (2006). Adaptive linear step-up procedures that control the false discovery rate. Biometrika.

[23] Uppal K., Walker D.I., Jones D.P. (2017). xMSannotator: An R package for network-based annotation of high-resolution metabolomics data. Anal. Chem..

[24] R: The R Project for Statistical Computing. https://www.r-project.org/.

[25] Smith C.A., O’Maille G., Want E.J., Qin C., Trauger S.A., Brandon T.R., Custodio D.E., Abagyan R., Siuzdak G. (2005). METLIN: A metabolite mass spectral database. Ther. Drug Monit..

[26] EUR-Lex, Access to European Union Law, 2002/657/EC. https://eur-lex.europa.eu/legal-content/EN/TXT/?qid=1585663763029&uri=CELEX:32002D0657.

[27] Hanahan D., Weinberg R.A. (2000). The Hallmarks of Cancer. Cell.

[28] Kroemer G., Pouyssegur J. (2008). Tumor Cell Metabolism: Cancer’s Achilles’ Heel. Cancer Cell.

[29] Romero-Garcia S., Moreno-Altamirano M.M.B., Prado-Garcia H., Sánchez-García F.J. (2016). Lactate Contribution to the Tumor Microenvironment: Mechanisms, Effects on Immune Cells and Therapeutic Relevance. Front Immunol..

[30] Warburg O. (1956). On the Origin of Cancer Cells. Science.

[31] Antognelli C., Talesa V.N. (2018). Glyoxalases in Urological Malignancies. Int. J. Mol. Sci..

[32] Bair W.B., Cabello C.M., Uchida K., Bause A.S., Wondrak G.T. (2010). GLO1 Overexpression in Human Malignant Melanoma. Melanoma Res..

[33] Filipp Fabian V., Ratnikov B., De Ingeniis J., Smith Jeffrey W., Osterman Andrei L., Scott David A. (2012). Glutamine-fueled mitochondrial metabolism is decoupled from glycolysis in melanoma. Pigment Cell Melanoma Res..

[34] De Ingeniis J., Ratnikov B., Richardson A.D., Scott D.A., Aza-Blanc P., De S.K., Kazanov M., Pellecchia M., Ronai Z., Osterman A.L. (2012). Functional Specialization in Proline Biosynthesis of Melanoma. PLoS ONE.

[35] Dillon B.J., Prieto V.G., Curley S.A., Ensor C.M., Holtsberg F.W., Bomalaski J.S., Clark M.A. (2004). Incidence and distribution of argininosuccinate synthetase deficiency in human cancers. Cancer.

[36] Locasale J.W., Grassian A.R., Melman T., Lyssiotis C.A., Mattaini K.R., Bass A.J., Heffron G., Metallo C.M., Muranen T., Sharfi H. (2011). Phosphoglycerate dehydrogenase diverts glycolytic flux and contributes to oncogenesis. Nat. Genet..

[37] Locasale J.W. (2013). Serine, glycine and the one-carbon cycle: Cancer metabolism in full circle. Nat. Rev. Cancer.

[38] Bessman S.P., Carpenter C.L. (1985). The Creatine-Creatine Phosphate Energy Shuttle. Annu. Rev. Biochem..

[39] de Santana-Filho A.P., Jacomasso T., Riter D.S., Barison A., Iacomini M., Winnischofer S.M.B., Sassaki G.L. (2017). NMR metabolic fingerprints of murine melanocyte and melanoma cell lines: Application to biomarker discovery. Sci. Rep..

[40] Granovsky M., Fata J., Pawling J., Muller W.J., Khokha R., Dennis J.W. (2000). Suppression of tumor growth and metastasis in Mgat5-deficient mice. Nat. Med..

[41] Dennis J.W., Laferté S. (1989). Oncodevelopmental Expression of—GlcNAcβ1–6Manα1-6Manβ1—Branched Asparagine-linked Oligosaccharides in Murine Tissues and Human Breast Carcinomas. Cancer Res..

[42] Sasai K., Ikeda Y., Fujii T., Tsuda T., Taniguchi N. (2002). UDP-GlcNAc concentration is an important factor in the biosynthesis of β1,6-branched oligosaccharides: Regulation based on the kinetic properties of N-acetylglucosaminyltransferase V. Glycobiology.

[43] Croze M.L., Soulage C.O. (2013). Potential role and therapeutic interests of myo-inositol in metabolic diseases. Biochimie.

[44] Novogrodskyl A., Nordenberg J. The Anti-Proliferative Effect of Lithium Chloride on Melanoma Cells and Its Reversion by Myo-Inositol. https://www.researchgate.net/figure/The-effect-of-myo-inositol-on-tumorigenicity-of-untreated-and-LiCI-pretreated-B-16_fig1_20126741.

[45] Ripps H., Shen W. (2012). Review: Taurine: A “very essential” amino acid. Mol. Vis..

[46] Liesenfeld D.B., Habermann N., Owen R.W., Scalbert A., Ulrich C.M. (2013). Review of Mass Spectrometry–Based Metabolomics in Cancer Research. Cancer Epidemiol. Biomark. Prev..

[47] Wu H., Xue R., Tang Z., Deng C., Liu T., Zeng H., Sun Y., Shen X. (2010). Metabolomic investigation of gastric cancer tissue using gas chromatography/mass spectrometry. Anal. Bioanal. Chem..

[48] SMPDB v.2.0, Purine Metabolism Pathway. http://smpdb.ca/view/SMP0000050.

[49] Sugimoto M., Wong D.T., Hirayama A., Soga T., Tomita M. (2010). Capillary electrophoresis mass spectrometry-based saliva metabolomics identified oral, breast and pancreatic cancer-specific profiles. Metabolomics.

[50] Wang X., Hu M., Liu M., Hu J. (2014). Metastatic Melanoma Induced Metabolic Changes in C57BL/6J Mouse Stomach Measured by 1H NMR Spectroscopy. Metabolomics (Los Angel).

[51] Galons J.P., Job C., Gillies R.J. (1995). Increase of GPC levels in cultured mammalian cells during acidosis. A 31P MR spectroscopy study using a continuous bioreactor system. Magn. Reson. Med..

[52] Holeček M. (2018). Branched-chain amino acids in health and disease: Metabolism, alterations in blood plasma, and as supplements. Nutr. Metab..

[53] 7-Hydroxy-6-methyl-8-ribityl lumazine-Biomarker|BiomarkerBase. https://www.biomarkerbase.com/7-hydroxy-6-methyl-8-ribityl-lumazine?utm_source=adis.

[54] SMPDB v.2.0, Riboflavin Metabolism Pathway. http://smpdb.ca/view/SMP0000070.

[55] Li X., Zhang A., Sun H., Liu Z., Zhang T., Qiu S., Liu L., Wang X. (2017). Metabolic characterization and pathway analysis of berberine protects against prostate cancer. Oncotarget.

[56] Wu H., Southam A.D., Hines A., Viant M.R. (2008). High-throughput tissue extraction protocol for NMR- and MS-based metabolomics. Anal. Biochem..

[57] Goodacre R., Broadhurst D., Smilde A.K., Kristal B.S., Baker J.D., Beger R., Bessant C., Connor S., Capuani G., Craig A. (2007). Proposed minimum reporting standards for data analysis in metabolomics. Metabolomics.

[58] Wei X., Shi X., Kim S., Zhang L., Patrick J.S., Binkley J., McClain C., Zhang X. (2012). Data preprocessing method for liquid chromatography-mass spectrometry based metabolomics. Anal. Chem..

[59] Cho H.W., Kim S.B., Jeong M.K., Park Y., Miller N.G., Ziegler T.R., Jones D.P. (2008). Discovery of metabolite features for the modelling and analysis of high-resolution NMR spectra., Discovery of metabolite features for the modelling and analysis of high-resolution NMR spectra. Int. J. Data Min. Bioinform..

[60] Craig A., Cloarec O., Holmes E., Nicholson J.K., Lindon J.C. (2006). Scaling and normalization effects in NMR spectroscopic metabonomic data sets. Anal. Chem..

[61] Pluskal T., Castillo S., Villar-Briones A., Orešič M. (2010). MZmine 2: Modular framework for processing, visualizing, and analyzing mass spectrometry-based molecular profile data. BMC Bioinform..

[62] Wen B., Mei Z., Zeng C., Liu S. (2017). metaX: A flexible and comprehensive software for processing metabolomics data. BMC Bioinform..

[63] Myers O.D., Sumner S.J., Li S., Barnes S., Du X. (2017). One Step Forward for Reducing False Positive and False Negative Compound Identifications from Mass Spectrometry Metabolomics Data: New Algorithms for Constructing Extracted Ion Chromatograms and Detecting Chromatographic Peaks. Anal. Chem..

[64] Du P., Kibbe W.A., Lin S.M. (2006). Improved peak detection in mass spectrum by incorporating continuous wavelet transform-based pattern matching. Bioinformatics.

