# Supplementary material for: Human Melanoma-Cell Metabolic Profiling: Identification of Novel Biomarkers Indicating Metastasis
Source: Int J Mol Sci. 2020 Mar 31;21(7):2436. doi: 10.3390/ijms21072436 (PMC7177954; doi:10.3390/ijms21072436)
Supplement: Supplementary file 1 [file ijms-21-02436-s001.zip › Kosmopoulou et al_Supplementary_Material_IJMS.docx]

**Supplementary Materials**

*Article*

**Human melanoma-cell metabolic profiling: identification of novel biomarkers indicating metastasis**

**Mariangela Kosmopoulou^1^, Aikaterini F. Giannopoulou^2^, Aikaterini Iliou^1^, Dimitra Benaki^1^, Aris Panagiotakis^1^, Athanassios D. Velentzas^2^, Eumorphia G. Konstantakou^3^, Issidora S. Papassideri^2^, Emmanuel Mikros^1,#,*^, Dimitrios J. Stravopodis^2,#,*^ and Evagelos Gikas^1,#,*^**

^1^ Section of Pharmaceutical Chemistry, Department of Pharmacy, School of Health Sciences, National and Kapodistrian University of Athens (NKUA), Athens 15701, Greece, mrgkosm@gmail.com (M.K.); katerinail@pharm.uoa.gr (A.I.); dbenaki@pharm.uoa.gr (D.B.); arispan@pharm.uoa.gr (A.P.)

^2^ Section of Cell Biology and Biophysics, Department of Biology, School of Science, National and Kapodistrian University of Athens (NKUA), Athens 15701, Greece, aigiann@biol.uoa.gr (A.F.G.); tveletz@biol.uoa.gr (A.D.V.); ipapasid@biol.uoa.gr (I.S.P.)

^3^ Harvard Medical School, Massachusetts General Hospital Cancer Center (MGHCC), Charlestown 021004, MA, USA, ekonstantakou@mgh.harvard.edu

^#^ These, senior authors, contributed equally to this work

**^*^** Correspondence: mikros@pharm.uoa.gr (E.M.); dstravop@biol.uoa.gr (D.J.S.); vgikas@pharm@uoa.gr (Ε.G.)

Received: date; Accepted: date; Published: date

**Abstract:** Melanoma is the most aggressive type of skin cancer, leading to metabolic rewiring and enhancement of metastatic transformation. Efforts to improve its early and accurate diagnosis are largely based on preclinical models and especially cell lines. Hence, we herein present a combinational NMR- and UHPLC-HRMS/MS-mediated untargeted metabolomic profiling of melanoma cells, to landscape metabolic alterations likely controlling metastasis. The cell lines WM115 and WM2664, which belong to the same patient, were examined, with WM115 being derived from a primary, pre-metastatic, tumor and WM2664 clonally expanded from lymph-node metastases. Metabolite samples were analyzed using NMR and UHPLC-HRMS. Multivariate statistical analysis of high resolution NMR and MS (positive and negative ionization) results was performed by PCA, PLS-DA and OPLS-DA, while metastasis-related biomarkers were determined on the basis of VIP lists, S-plots and student’s t-tests. ROC curves of NMR and MS data revealed significantly differentiated metabolite profiles for each cell line, with WM115 being mainly characterized by upregulated levels of phosphocholine, choline, guanosine and inosine. Interestingly, WM2664 showed notably increased contents of hypoxanthine, myo-inositol, glutamate, organic acids, purines, pyrimidines, AMP, ADP, ATP and UDP(s), thus indicating the critical roles of purine, pyrimidine and amino acid metabolism during human melanoma metastasis.

**Keywords:** Biomarker; Cancer; Melanoma; Metabolomics; Metastasis; MS; NMR

**
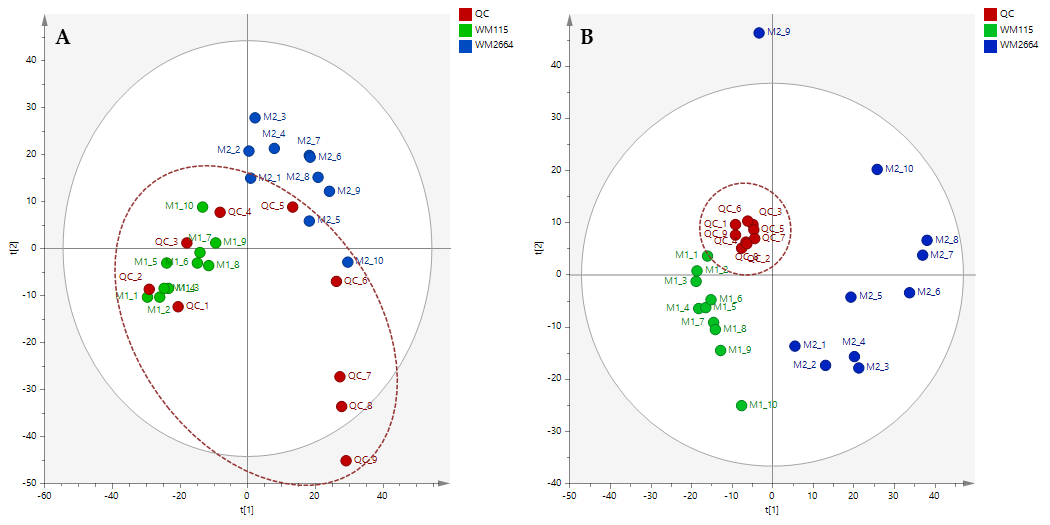
**

**Figure S1.** QCs were analyzed in order to investigate the instrument drift. (**A**) A poor clustering of QCs is observed in the scores plot of the PCA, indicating an instrument drift. (**B**) After a QC-robust spline batch correction (QC-RSC), using the peak based normalization in MetaX, feature distribution is reduced and QCs are tightly clustered.

**
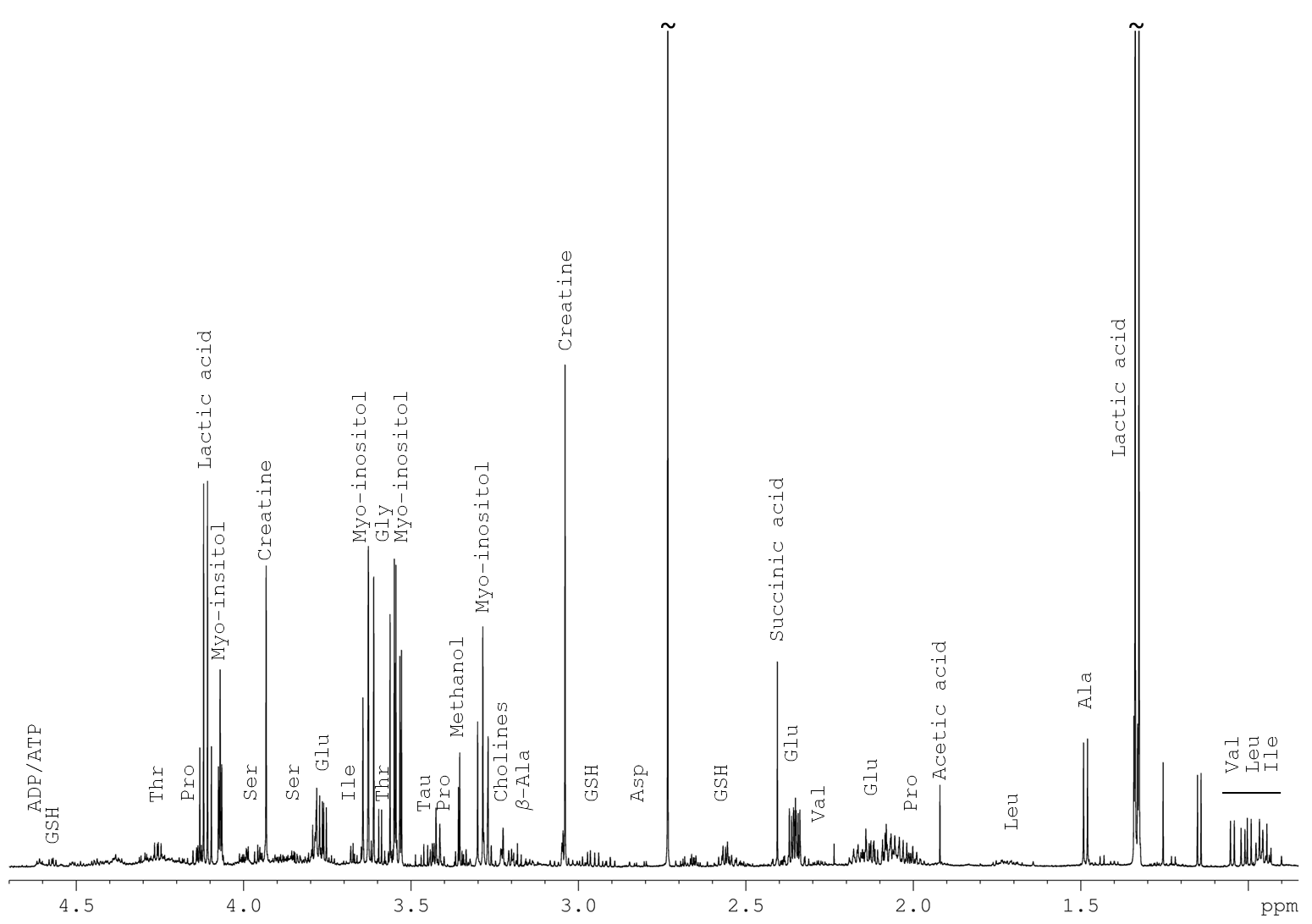

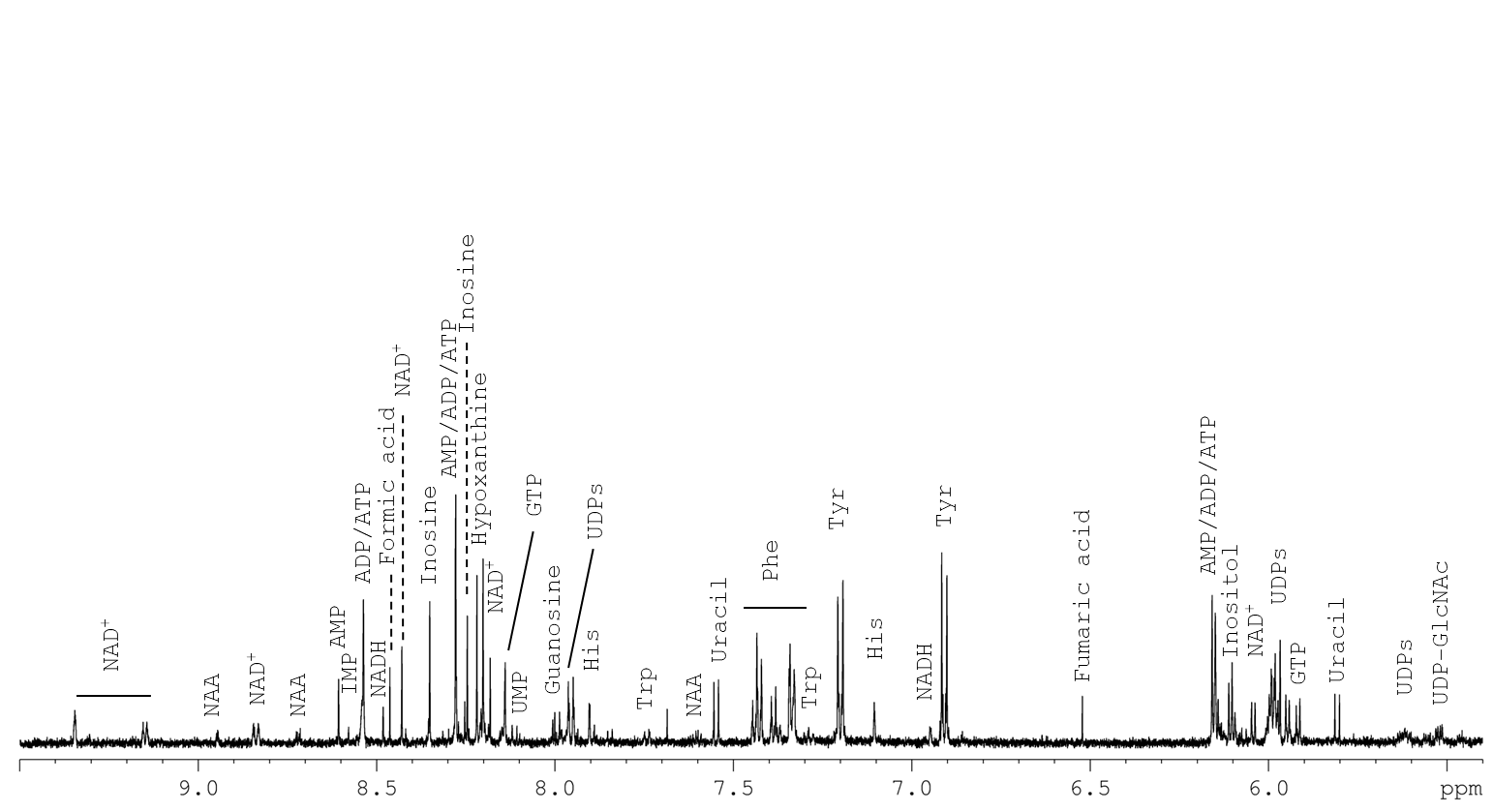
**

**Figure S2.** ^1^H NMR spectrum of the WM2664 -human- metastatic melanoma cells, with the identified metabolites.

**Table S1.** ^1^H Chemical shifts of the metabolites identified through the analysis of NMR spectra derived from WM115 (primary) and MW2664 (metastatic) melanoma-cell polar extracts

| **Category** | **No.** | **Metabolite** | **δ_H_ – Multiplicity**^1^ |
| --- | --- | --- | --- |
| Amino acids | 1 | Alanine (Ala) | 3.79 q; **1.49** d |
|  | 2 | *β*-Alanine (*β*-Ala) | **3.18** t; 2.56 t |
|  | 3 | Aspartic acid (Asp) | **2.82** dd |
|  | 4 | Glutamic acid (Glu) | 3.76 q; 2.37 m ; **2.34** m; 2.13 t; 2.06 m |
|  | 5 | Glutathione (GSH) | **4.57** q; 3.79d; 3.78t; 3.77d; 2.98q; 2.93q |
|  | 6 | Glycine (Gly) | **3.56** s |
|  | 7 | Histidine (His) | 7.89 d; **7.10** s |
|  | 8 | Isoleucine (Ile) | 3.68 d; **1.01** d; 0.94 t |
|  | 9 | Leucine (Leu) | 3.74 q ; 1.72 m; **0.97** d; 0.96 d |
|  | 10 | Phenylalanine (Phe) | **7.43** t; **7.38** t; **7.34** d; 4.00 q; 3.14 q |
|  | 11 | Proline (Pro) | **4.14** q; 3.43 m; 3.34 m; 2.35 m; 2.06 m; 1.99 m |
|  | 12 | Serine (Ser) | 4.00 q; **3.95** q; 3.85 q |
|  | 13 | Taurine (Tau) | 3.43 t; **3.26** t |
|  | 14 | Threonine (Thr) | 4.25 q; **3.59** d; 1.33 d |
|  | 15 | Tryptophan (Trp) | **7.74** d; 7.33 s; 7.29 t |
|  | 16 | Tyrosine (Tyr) | **7.20** d; 6.91 d; 3.95 q |
|  | 17 | Valine (Val) | 3.62 d; 2.28 m ; **1.05** d; 0.99 d |
| Alpha amino acids | 18 | Creatine | 3.93 s ; **3.04** s |
|  | 19 | Creatine phosphate | **3.96** s; 3.05 s |
|  | 20 | Creatinine | **4.06** s; 3.05 s |
| Organic acids | 21 | Acetic acid | **1.92** s |
|  | 22 | Citric acid | 2.64 d |
|  | 23 | Formic acid) | **8.46** s |
|  | 24 | Fumaric acid | **6.52** s |
|  | 25 | Lactic acid | 4.11 q; **1.33** d |
|  | 26 | Malic acid | 4.30 dd; 2.67 dd; 2.37 dd |
|  | 27 | Succinic acid | **2.41** s |
| Cholines | 28 | Choline | **3.207** s |
|  | 29 | O-Acetylcholine | 4.55 m ; **3.23** s; 2.14 s |
|  | 30 | O-Phosphocholine | **3.2** s |
|  | 31 | sn-Glycero-3-phosphocholine | **3.23** s |
| Purines | 32 | AMP | **8.61** s; 8.28 s; 6.145 d; 4.51 q |
|  | 33  34 | ADP &  ATP | **8.54** s; 8.28 s; 6.15 d |
|  | 35 | Guanosine | **8.01** s |
|  | 36 | GTP | 8.14 s; 5.95 d |
|  | 37 | Hypoxanthine | **8.22** s; 8.20 s |
|  | 38 | Inosine | **8.35** s; 8.245 s; 6.11 d |
|  | 39 | IMP | **8.58** s; 8.25 s |
|  | 40 | NAD^+^ | **8.43** s; 8.18 s |
|  | 41 | NADH | **8.48** s ; 8.24 s; 6.9 bs |
| Pyrimidines | 42 | Uracil | 7.55 d; **5.81** d |
|  | 43 | UDP-N-Acetylglucosamine  (UDP-GlcNAc) | 7.95 d; **5.52** q; 2.1 s |
|  | 44  45  46 | UDP-Galactose &  UDP-Glucose &  UDP-N-Acetylgalactose | **5.64-5.60** m |
|  | 47 | UDP-N-Acetylgalactosamine | **5.55** d |
|  | 48 | UMP | **8.11** d |
| Other | 49 | Myo-inositol | **4.07** t; 3.63 t; 3.54 q; 3.29 t |
|  | 50 | Niacinamide (NAA) | **8.94** dt; 8.71 dd |
| ^1^s: singlet; d: doublet; t: triplet; q: quartiplet;m: multiplet; dd; doublet of doublets; dt: doublet of triplets; bs: broad singlet | | | |

**Table S2.** Features contributing to the separation of WM115 (primary) and WM2664 (metastatic) -human- melanoma cells (t-test, FDR and S-plot)

| (-) ESI | | | | (+) ESI | | | |
| --- | --- | --- | --- | --- | --- | --- | --- |
| m/z | **Retention time** (t_R_) | **P value** (t-test) | **q value** (FDR  Q = 5%) | **m/z** | **Retention time** (t_R_) | **P value** (t-test) | **q value** (FDR  Q = 5%) |
| 118.9312 | 0.68 | 4.49e-013 | 1.98e-012 | 125.9860 | 21.69 | 0.00618 | 0.00584 |
| 282.0839 | 1.13 | 1.50e-013 | 9.09e-013 | 137.0456 | 1.20 | 4.87e-007 | 0.000005 |
| 365.0495 | 1.17 | 1.65e-013 | 9.09e-013 | 171.1491 | 2.39 | 0.00977 | 0.00839 |
| 328.0892 | 1.14 | 6.77e-013 | 2.49e-012 | 310.8290 | 0.74 | 0.000003 | 0.000012 |
| 633.1295 | 1.17 | 1.24e-012 | 3.90e-012 | 97.9685 | 21.50 | 0.0145 | 0.0114 |
| 133.0147 | 0.74 | 3.33e-011 | 9.19e-011 | 110.0598 | 0.95 | 0.00402 | 0.00520 |
| 611.1432 | 0.99 | 8.31e-011 | 2.04e-010 | 123.0550 | 1.04 | 0.00447 | 0.00520 |
| 564.7884 | 0.73 | 1.84e-009 | 3.70e-009 | 137.0456 | 0.86 | 0.000030 | 0.000094 |
| 504.7678 | 0.65 | 6.00e-009 | 1.10e-008 | 139.9878 | 21.52 | 0.0196 | 0.0143 |
| 320.0621 | 0.85 | 1.29e-008 | 2.19e-008 | 150.0582 | 0.86 | 0.00495 | 0.00520 |
| 252.9519 | 0.92 | 3.57e-008 | 5.25e-008 | 166.0862 | 2.05 | 0.000736 | 0.00173 |
| 422.0868 | 0.99 | 3.91e-008 | 5.25e-008 | 176.8940 | 0.68 | 0.0413 | 0.0260 |
|  |  |  |  | 182.0812 | 0.94 | 0.00152 | 0.00288 |
|  |  |  |  | 79.0208 | 0.85 | 0.00187 | 0.00294 |
|  |  |  |  | 86.0960 | 1.23 | 0.0306 | 0.0206 |

**Table S3.** Workflow and parameters used in MZmine 2.31, for targeted metabolite screening

| Workflow | Parameters MZmine 2.31 | | | |
| --- | --- | --- | --- | --- |
| Peak Detection - Targeted Peak Detection | Intensity tolerance = 50% | Noise level = 1.0E4 | m/z tolerance = 5 ppm | RT tolerance = 16 min |
| Alignment - Join Aligner | m/z tolerance = 5 ppm | RT tolerance = 0.2 min | Weight for m/z = 1 | Weight for RT = 1 |
| Filtering - Duplicate Peak Filter | m/z tolerance = 5 ppm | RT tolerance = 0.15 min | Intensity tolerance = 50% |  |

**Table S4.** (**A**) Mass spectrometer operating conditions in positive and negative ion mode. (**B**) Pre-processing workflow and parameters used in the MZmine 2.31 and MetaX

| **(Α) Parameters** |  | | | **Mass spectrometer operating conditions** | | | | | | |
| --- | --- | --- | --- | --- | --- | --- | --- | --- | --- | --- |
|  | | **(+) ESI** | | | | **(-) ESI** | | | | |
| Capillary temperature |  | | | 356 °C | | 356 °C | | | | |
| Capillary voltage |  | | | 20 V | | -60 V | | | | |
| Tube lens |  | | | -49 V | | 110 V | | | | |
| Source voltage |  | | | 3.10 kV | | 3.50 kV | | | | |
| Sheath gas flow |  | | | 30 arb. units | | 30 arb. units | | | | |
| Aux gas flow |  | | | 10 arb. units | | 10 arb. units | | | | |
| **(Β1) Workflow** |  | | | **Parameters MZmine 2.31** | | | | | | |
| Peak detection - Mass detection | Detector = centroid | | | Noise level = 1.0E4 |  | |  |  | | |
| Peak detection - ADAP chromatogram builder | Min group size in # of scans = 3.00 | | | Group intensity threshold = 5.0E3 | Min highest intensity = 2.0E3 | | m/z tolerance = 5 ppm |  | | |
| Peak detection - Chromatogram deconvolution (Wavelets ADAP) | S/N threshold = 10 | | | S/N estimator = Intensity window S/N | Min feature height = 10 | | Area threshold = 10 | Peak duration range = 0.03-0.30 | | |
| Isotopic peak grouper | m/z tolerance = 10 ppm | | | RT tolerance = 0.2 min |  | |  |  |  |  |
| Alignment - Join aligner | m/z tolerance = 10 ppm | | | RT tolerance = 0.15 min | Weight for m/z = 1 | | Weight for RT = 1 |  |  |  |
| Gap filling - Peak finder | m/z tolerance = 5 ppm | | | RT tolerance = 0.15 min | Intensity tolerance = 50% | |  |  |  |  |
| **(B2) Parameters MetaX** | | | |  |  | |  |  |  |  |
| Miss value ratio in true samples | | | 30% | | | | | | |  |
| Miss value ratio in QC samples | | | 80% | | | | | | |  |
| Coefficient variation threshold | | | 0.3 | | | | | | |  |
| Missing value imputation method | | | KNN | | | | | | |  |
| Scaling method | | | Pareto | | | | | | |  |
| Transformation method | | | None | | | | | | |  |
| Normalization method | | | QC-RSC | | | | | | |  |
