# Supplementary figures and images for: Human Melanoma-Cell Metabolic Profiling: Identification of Novel Biomarkers Indicating Metastasis
Source: Int J Mol Sci. 2020 Mar 31;21(7):2436. doi: 10.3390/ijms21072436 (PMC7177954; doi:10.3390/ijms21072436)

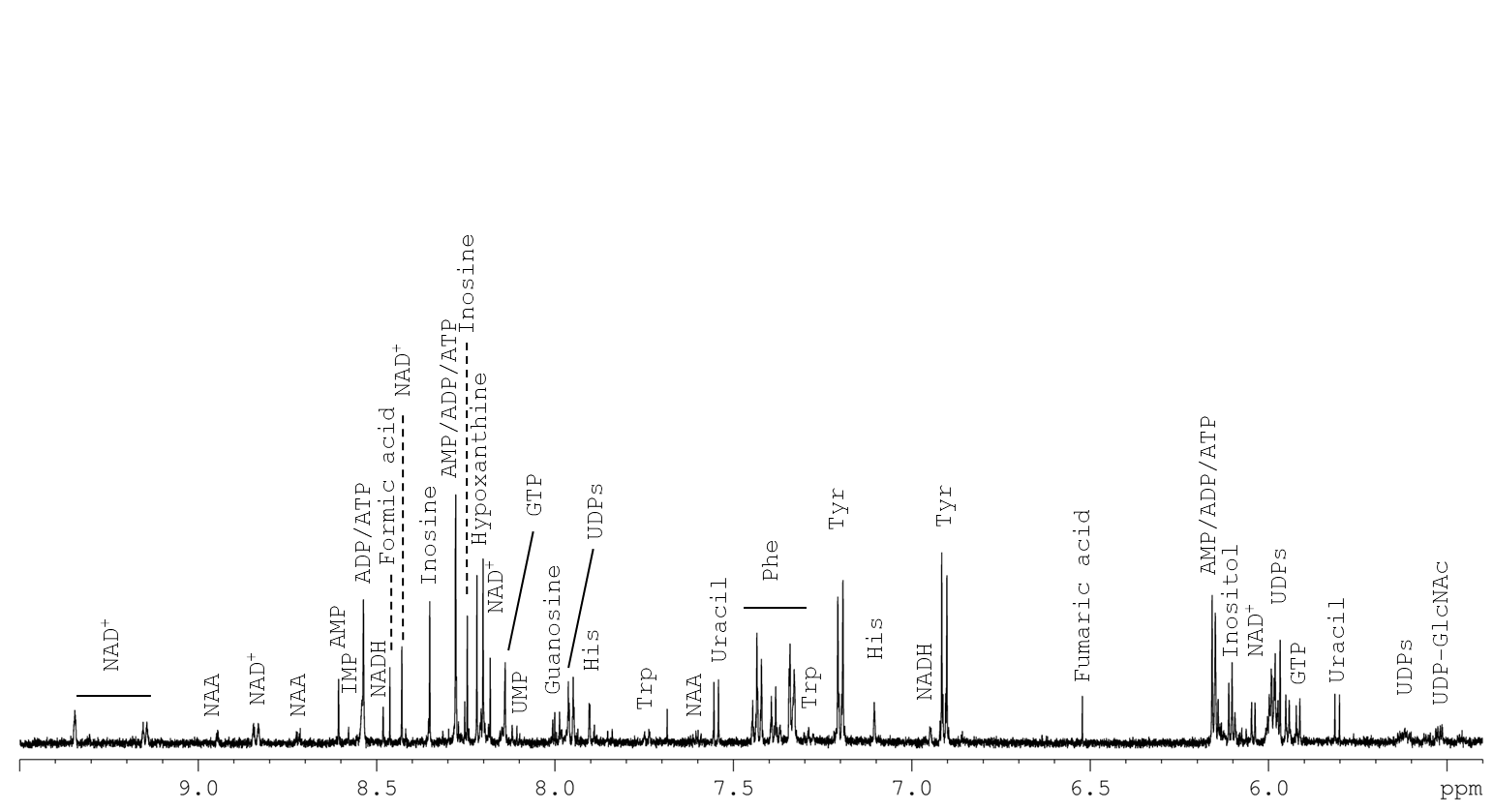

Supplement: Supplementary file 1 [file ijms-21-02436-s001.zip › Figure S2(2).tif]

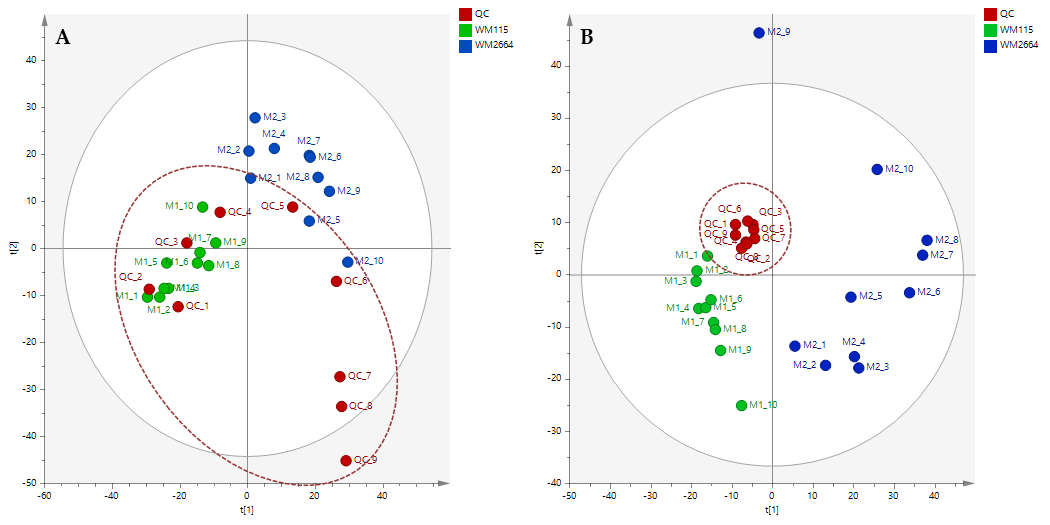

Supplement: Supplementary file 1 [file ijms-21-02436-s001.zip › Figure S1.tif]

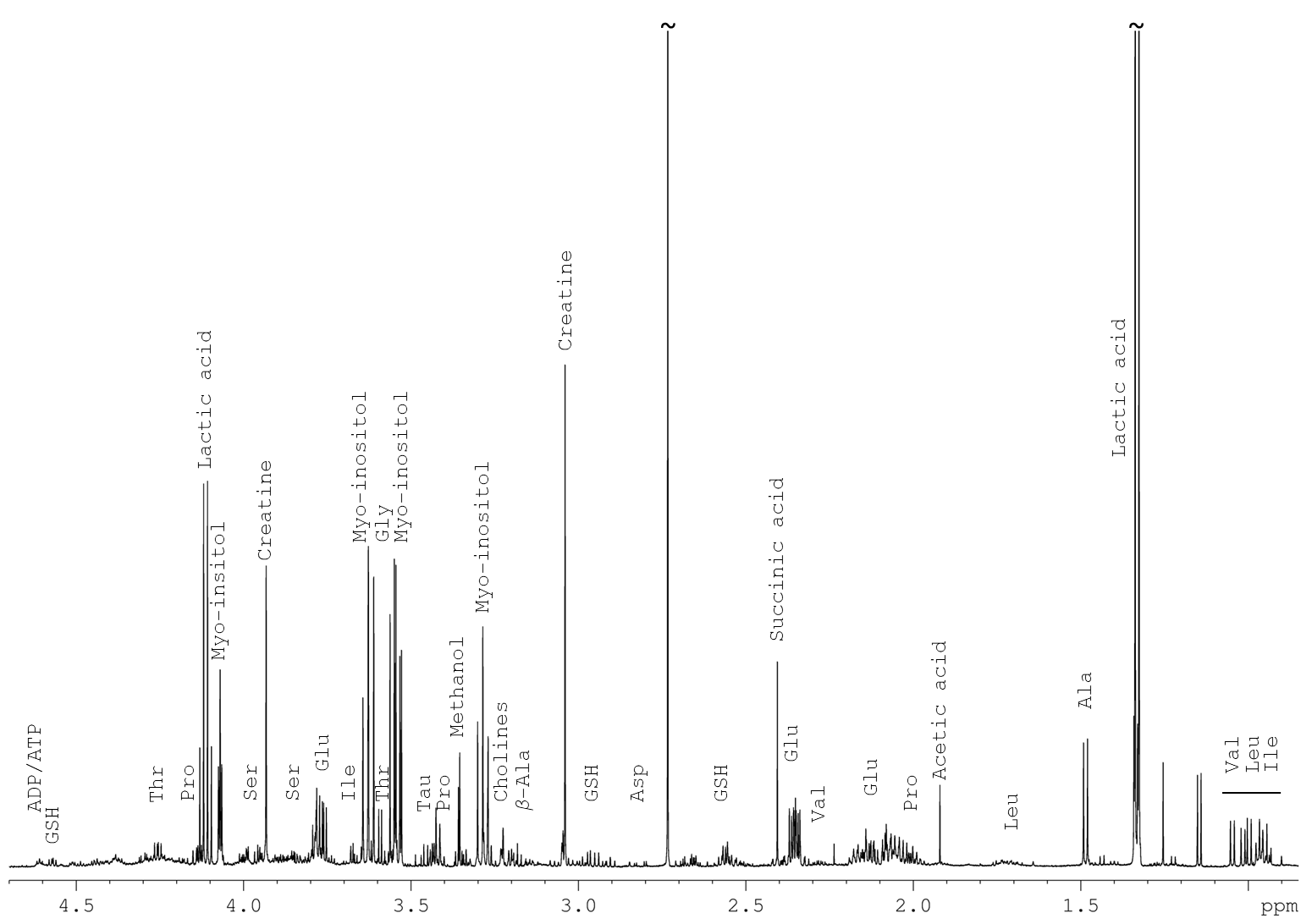

Supplement: Supplementary file 1 [file ijms-21-02436-s001.zip › Figure S2(1).tif]
